# Telomere‐to‐telomere genome assembly reveals insights into the adaptive evolution of herbivore‐defense mediated by volatile terpenoids in *Oenanthe javanica*

**DOI:** 10.1111/pbi.70062

**Published:** 2025-03-20

**Authors:** Kai Feng, Jia‐Lu Liu, Nan Sun, Zi‐Qi Zhou, Zhi‐Yuan Yang, Hui Lv, Cheng Yao, Jin‐Ping Zou, Shu‐Ping Zhao, Peng Wu, Liang‐Jun Li

**Affiliations:** ^1^ College of Horticulture and Landscape Architecture Yangzhou University Yangzhou China; ^2^ Key Laboratory of Biobreeding for Specialty Horticultural Crops of Jiangsu Province, Joint International Research Laboratory of Agriculture and Agri‐Product Safety of Ministry of Education of China Yangzhou University Yangzhou China

**Keywords:** *Oenanthe javanica* (Blume) DC., T2T genome, whole‐genouplicate, terpene synthase, plant–herbivore interaction

## Abstract

Releasing large quantities of volatiles is a defense strategy used by plants to resist herbivore attack. *Oenanthe javanica*, a perennial herb of the Apiaceae family, has a distinctive aroma due to volatile terpenoid accumulation. At present, the complete genome and genetic characteristics of volatile terpenoids in *O. javanica* remain largely unclear. Here, the telomere‐to‐telomere genome of *O. javanica*, with a size of 1012.13 Mb and a contig N50 of 49.55 Mb, was established by combining multiple sequencing technologies. Comparative genome analysis revealed that *O. javanica* experienced a recent species‐specific whole‐genome duplication event during the evolutionary process. Numerous gene family expansions were significantly enriched in the terpenoid biosynthesis process, monoterpenoid, and diterpenoid biosynthesis pathways, which resulted in abundant volatile substance accumulation in *O. javanica.* The volatile terpenoids of *O. javanica* showed repellent effects on herbivores. Terpenoid biosynthesis was activated by wounding signals under exogenous stimuli. The TPS gene family was significantly expanded in *O. javanica* compared to those in other species, and the members (*OjTPS1*, *OjTPS3*, *OjTPS4*, *OjTPS5*, *OjTPS7*, *OjTPS16*, *OjTPS18*, *OjTPS30* and *OjTPS58*) responsible for different terpenoid biosynthesis were functionally characterized. These results reveal the genome evolution and molecular characteristics of volatile terpenoids in the process of plant–herbivore interactions. This study also provides genomic resources for genetic and molecular biology research on *O. javanica* and other plants.

## Introduction

The genus *Oenanthe* L. contains over 30 species that are widely distributed around the world. *Oenanthe javanica* (Blume) DC. is a perennial herb in the Apiaceae family (Wang *et al*., 2022). In China, *O. javanica* is a medicinal food and homologous species that is consumed as a traditional Chinese medicine and edible vegetable (Feng *et al*., 2018; Lu and Li, 2019). Bioactive substances confer *O. javanica* with therapeutic potential for ethanol‐induced liver damage, jaundice and inflammation (Gam *et al*., 2022; Jo *et al*., 2022). As an edible plant, *O. javanica* has a unique flavour due to its rich volatile substances (Feng *et al*., 2023), and it is used as a popular food additive and spice in Southeast Asia (Kongkachuichai *et al*., 2015; Seo and Baek, 2005).

Wild *O. javanica* usually grows in wet areas, such as wetlands, swamps, riverbanks and lakesides, where it experiences less herbivore infestation during growth (Wang *et al*., 2022). Volatile substances in plants are used as chemical signals for resisting herbivores and attracting pollinators (Richards *et al*., 2015). *Coriandrum sativum*, another Apiaceae plant, also exhibits a distinctive flavour. The characteristics of *C. sativum* that cause controversial feelings were deciphered based on the genome assembly (Song *et al*., 2019). Terpenoids are the main volatile components in *O. javanica* (Deng *et al*., 2003; Seo and Baek, 2005). Some rate‐limiting enzymes and regulatory genes related to volatile terpenoids have been identified based on biochemistry and molecular biology techniques (Feng *et al*., 2022, 2024). However, the characteristics of volatile terpenoids in *O. javanica* from the perspectives of genetics, evolution and genomics are poorly understood.

To date, chromosome‐level genomes have been reported in several Apiaceae plants, including *Daucus carota* (Iorizzo *et al*., 2016), *Angelica sinensis* (Han *et al*., 2022), *C. sativum* (Song *et al*., 2019), *Cryptotaenia japonica* (Liu *et al*., 2024) and *Oenanthe sinensis* (Liu *et al*., 2023). Assembled genomes have provided genetic information for investigating the evolution and mechanism of characteristic substances in Apiaceae plants (Coe *et al*., 2023; Song *et al*., 2021). However, due to the sequence complexity of repetitive regions in centromeres and telomeres, there are still many gaps and missing sequences in these genomes (Nurk *et al*., 2022). In 2021, a draft genome was published for *O. javanica via* HiSeq 2000 sequencing technology, and the response mechanism to water stress was investigated based on multi‐omics analysis (Liu *et al*., 2021). However, the draft genome assembly failed to anchor to the pseudochromosome due to the absence of high‐throughput chromosome conformation capture (Hi‐C) technology. Many genome gaps are still unknown due to the sequencing and assembly methods. An incomplete genome limits genetic and molecular mechanism research on *O. javanica*, and a high‐quality genome is indispensable.

With the development of long‐read sequencing technology and computational algorithms, a telomere‐to‐telomere (T2T) genome, also known as a gapless genome, has made it possible to obtain the complete genetic information of genomes (Kille *et al*., 2022; Logsdon *et al*., 2020). T2T genomes decipher the genome sequences of highly repetitive regions, centromeres and telomeres on chromosomes to obtain the complete genome (Nurk *et al*., 2022). In recent years, T2T genomes have been reported in many model plants and horticultural crops, such as Arabidopsis (Naish *et al*., 2021), maize (Chen *et al*., 2023), watermelon (Deng *et al*., 2022) and *Brassica rapa* (Zhang *et al*., 2023). At present, only the T2T genome of *D. carota* has been published in the Apiaceae family (Wang *et al*., 2023).

Here, the first T2T genome was constructed for *O. javanica* by combining ultra‐long Oxford Nanopore Technology (ONT) and PacBio high‐fidelity (Pacbio HiFi) sequencing (Cheng *et al*., 2021; Jain *et al*., 2018). Karyotype analysis was conducted on *O. javanica*, and the gap‐free genome assembly was anchored to 21 pseudochromosomes using Hi‐C technology. The centromeres and telomeres of the *O. javanica* genome were identified on chromosomes, resulting in a genome with high continuity, accuracy and integrity. The adaptive evolution of herbivore defense mediated by volatile terpenoids in *O. javanica* was deciphered from the aspects of whole‐genome duplication (WGD), gene expansion and terpene synthase (TPS) family functions based on the genome. These results offer complete genomic information for *O. javanica* and insights into the herbivore defense mechanism of Apiaceae plants.

## Results

### T2T genome assembly of *O. javanica*

**Figure 1:**
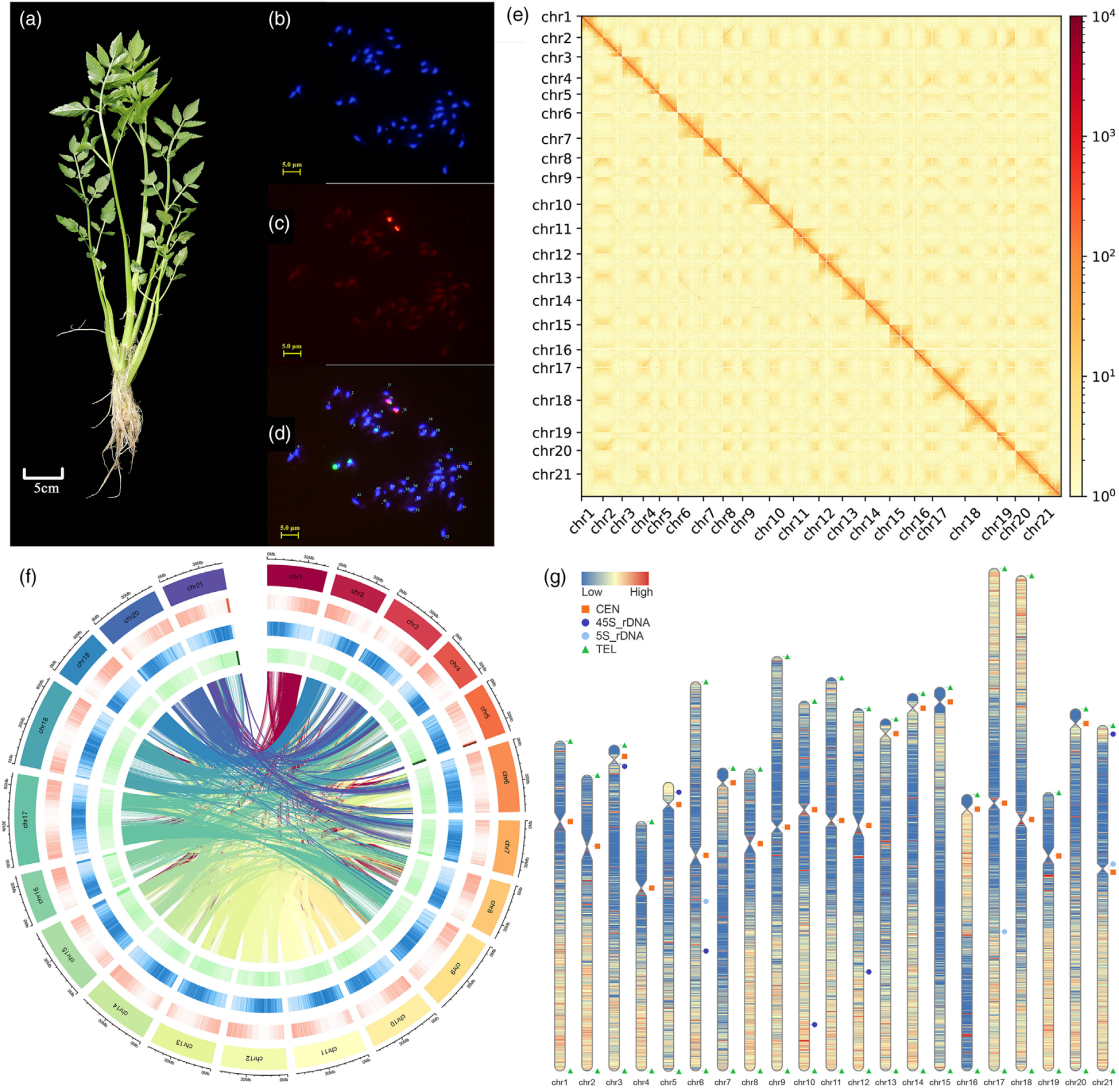
Phenotypes, karyotype analysis, fluorescence *in situ* hybridization (FISH) and Hi‐C analysis of *Oenanthe javanica*. (a) *Oenanthe javanica* variety ‘Fuqin No. 1’. (b–d) Karyotype analysis and FISH assays of *O. javanica*. (e) Hi‐C map of *O. javanica*. (f) Circos plot (circles from inside to outside represent the GC content, repeat density and gene density). (g) Telomere and centromere distribution on *O. javanica* chromosomes.

*Oenanthe javanica* cultivar ‘Fuqin No. 1’ was used as the plant material for genome sequencing and assembly (Figure 1a). A total of 21 pairs of chromosomes were identified in *O. javanica* based on karyotype analysis and fluorescence *in situ* hybridization (Figure 1b–d). The *O. javanica* genome was estimated to be 951.53 Mb, according to the genome survey (Figure S1 and Table S1). The *O. javanica* genome was sequenced using ONT ultra‐long, Pacbio HiFi and next‐generation platforms, resulting in 160.38 (~140.17×), 90.46 (~86.37×) and 99.74 Gb (~96.96×) pass reads, respectively (Tables S2–S4). The *O. javanica* genome was assembled using the ONT ultra‐long and Pacbio HiFi data and successfully anchored to 21 pseudochromosomes based on the Hi‐C assembly (Figure 1e and Tables S5 and S6). The genome was further polished, and the detected gaps were filled and corrected (Table S7). Finally, a T2T genome was obtained for *O. javanica* with no gap, showing a size of 1012.13 Mb and a contig N50 of 49.55 Mb (Table 1). To determine the genome consistency, the clean reads from next‐generation sequencing (NGS), ONT ultra‐long sequencing and Pacbio HiFi sequencing were mapped (mapping rate > 99.62%) to the T2T genome (Table S8). The quality value (QV) of each chromosome was determined, and the QV of the whole genome was 42.66 (Table S9). Analysis showed that 98.5% of benchmarking universal single‐copy orthologs (BUSCOs) were complete (Table S10). These results indicate that the *O. javanica* T2T genome was a gapless genome with high consistency, accuracy and completeness.

**Table 1:** Summary of T2T gap‐free genome assembly of *Oenanthe javanica*

Parameters	*O. javanica* T2T
Genome size (Mb)	1012.13
Contig N50 (Mb)	49.55
Contig number	21
QV value	42.66
Gaps	0
Number of telomeres	41
Number of centromeres	21
BUSCOs (%)	98.5

### Identification of telomeres and centromeres

Telomere and centromere region identification posed challenges due to the high density of short tandem repeats and low gene density. The gene density, GC content and repeat sequence density were represented in a circos plot (Figure 1f). To further investigate the genome structure, telomeres and centromeres were identified in the current T2T genome (Figure 1g). A total of 41 telomeres were detected using repetitive sequences (CCCTAAA/TTTAGGG at 5′ end/3′ end) as queries. Except for chr 5, all other chromosomes had two telomeres at each chromosome end (Table S11). The centromeric regions were identified based on centromeric tandem repeat sequences (Deng *et al*., 2022). A total of 21 centromeres were identified, and the distribution on each chromosome is shown in Table S12.

### Genome annotation

The repetitive sequences, noncoding RNA and gene structures and functions were annotated in the *O. javanica* T2T genome. The repeat sequences were annotated using multiple methods. Through integration and redundancy of the predicted results, 656.29 Mb transposable elements (TEs) were obtained. The TE sequences accounted for 64.84% of the *O. javanica* T2T genome, with long‐terminal repeats (LTR) having the highest proportion (49.55%) (Table S13). The tandem repeats (8.19%) were also annotated, including 0.538% microsatellites, 4.861% minisatellites and 5.597% satellites (Table S14). Although noncoding RNA (ncRNA) cannot be translated into proteins, it has important biological functions in plants. In this study, 264 miRNAs with an average length of 132 bp were annotated in the genome. The number of tRNAs, rRNAs and snRNAs was determined to be 933, 3070 and 1169, respectively (Table S15). The gene structure of *O. javanica* T2T genome was analysed using multiple methods, including *de novo* prediction, transcriptome prediction and homology (*D. carota*, *A. graveolens*, *C. sativum* and *A. thaliana*) prediction. A total of 65,763 protein‐coding genes were predicted in the *O. javanica* T2T genome, and the average mRNA and coding sequence (CDS) lengths were 4448.34 bp and 1103.83 bp, respectively (Table S16). The gene, CDS, exon and intron length distributions in *O. javanica* were similar to those in *D. carota*, *A. graveolens* and *C. sativum* (Figure S2). Gene function annotation was performed using different databases, such as Kyoto Encyclopedia of Genes and Genomes (KEGG), nonredundant protein (Nr), Gene Ontology (GO), Eukaryotic Orthologous Genes (KOG), Pfam and Interpro, as references, and 65,763 genes (92.72%) were annotated (Figure S3 and Table S17).

### Comparative genomics and WGD analysis

**Figure 2:**
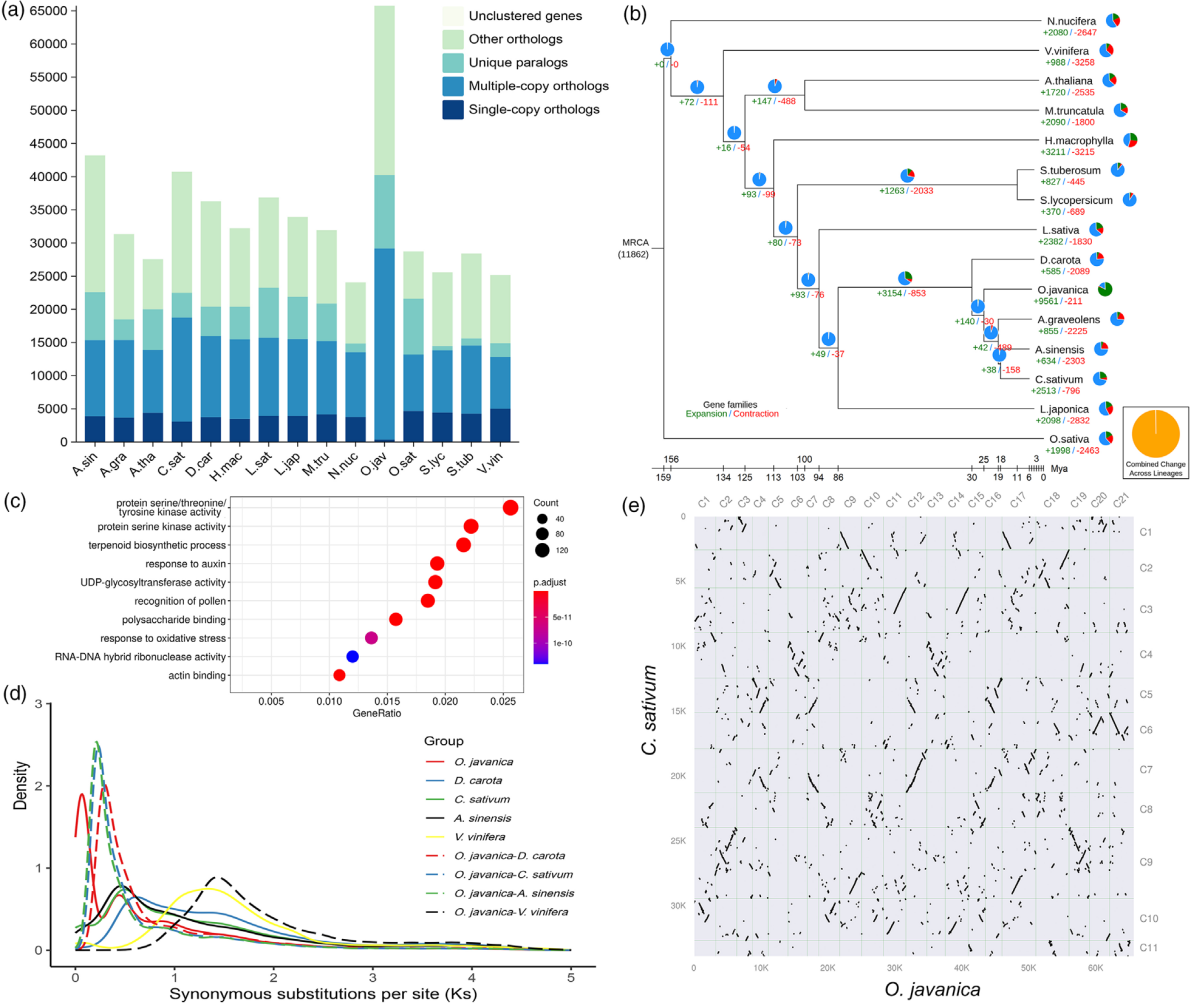
Comparative genomic analysis of *Oenanthe javanica* and other plants. (a) Statistics of homologous genes among plants. (b) Divergence time and expansion and contraction of gene families among species. (c) GO enrichment analysis of expanded gene families in the *O. javanica* T2T genome. (d) Synonymous substitutions per synonymous site (*Ks*) analysis of paralogs and orthologs of *O. javanica* and four other species. (e) Syntenic genes between the *O. javanica* and *Co*. *sativum* genomes.

*O. javanica* (O.jav) and 14 other species were selected to conduct the comparative genomic analysis, including *A. sinensis* (A.sin), *A. graveolens* (A.gra), *A. thaliana* (A.tha), *C. sativum* (C.sat), *D. carota* (D.car), *Hydrangea macrophylla* (H.mac), *Lactuca sativa* (L.sat), *Lonicera japonica* (L.jap), *Medicago truncatula* (M.tru), *Nelumbo nucifera* (N.nuc), *Oryza sativa* (O.sat), *Solanum lycopersicum* (S.lyc), *Solanum tuberosum* (S.tub) and *Vitis vinifera* (V.vin). A total of 64,305 orthologous gene families were identified, and the gene number in *O. javanica* was significantly higher than in other species. Among these, 9093 unique gene families were identified from *O. javanica*, containing 11,087 paralogs (Figure 2a and Table S18). A total of 9561 gene families expanded during the evolution of *O. javanica*, which was significantly higher than that in other species (Figure 2b). Gene family expansion enables plants to further adapt to the environment during the evolutionary process (Moore and Purugganan, 2005). GO and KEGG enrichment analysis showed that numerous expanded gene families were significantly enriched in the terpenoid biosynthesis process, monoterpenoid biosynthesis and diterpenoid biosynthesis, explaining the biosynthesis and accumulation of rich fragrant substances in *O. javanica* (Figure 2c and Tables S19 and S20).

To further investigate the evolution and genome expansion, WGD was analysed using the distributions of synonymous substitutions per synonymous site (*Ks*) of collinear gene pairs (Figure 2d). Multiple peaks were detected in *O. javanica* using *Ks* analysis, and the peaks at ~0.45 and ~0.9 were consistent with the previously reported α and ω WGD events in Apiaceae (Song *et al*., 2021). Notably, the peak at ~0.08 indicated that a species‐specific WGD event occurred at 3.6–6.5 Mya, which was not detected in other Apiaceae plants. To identify the key gene duplications in the species‐specific WGD event of the evolutionary process, the collinear gene pairs (*Ks* < 0.1) were extracted and visualized on chromosomes (Figure S4). The enrichment analysis of collinear gene pairs (*Ks* < 0.1) was also performed to identify the key genes and pathways in the species‐specific WGD event (Figures S5 and S6 and Tables S21 and S22). Collinearity and synteny analyses were conducted in *O. javanica* and other Apiaceae plants (*C. sativum* and *D. carota*) to confirm the species‐specific WGD events (Figure S7). The syntenic depth ratio value of *O. javanica* to *C*. *sativum* and *O. javanica* to *D. carota* was 2:1 (Figure 2e and Figure S8). The karyotype evolution of Apiaceae plants from ancestral eudicot karyotypes (AEK) was investigated, and it showed that chromosome rearrangements occurred in the current Apiaceae plant genome (Figure S9). These results suggest that the species‐specific WGD event endowed *O. javanica* with a unique aroma and adaptability to waterlogging, which are not present in other Apiaceae plants.

### Repellent effects of *O. javanica* volatiles on herbivorous insects

**Figure 3:**
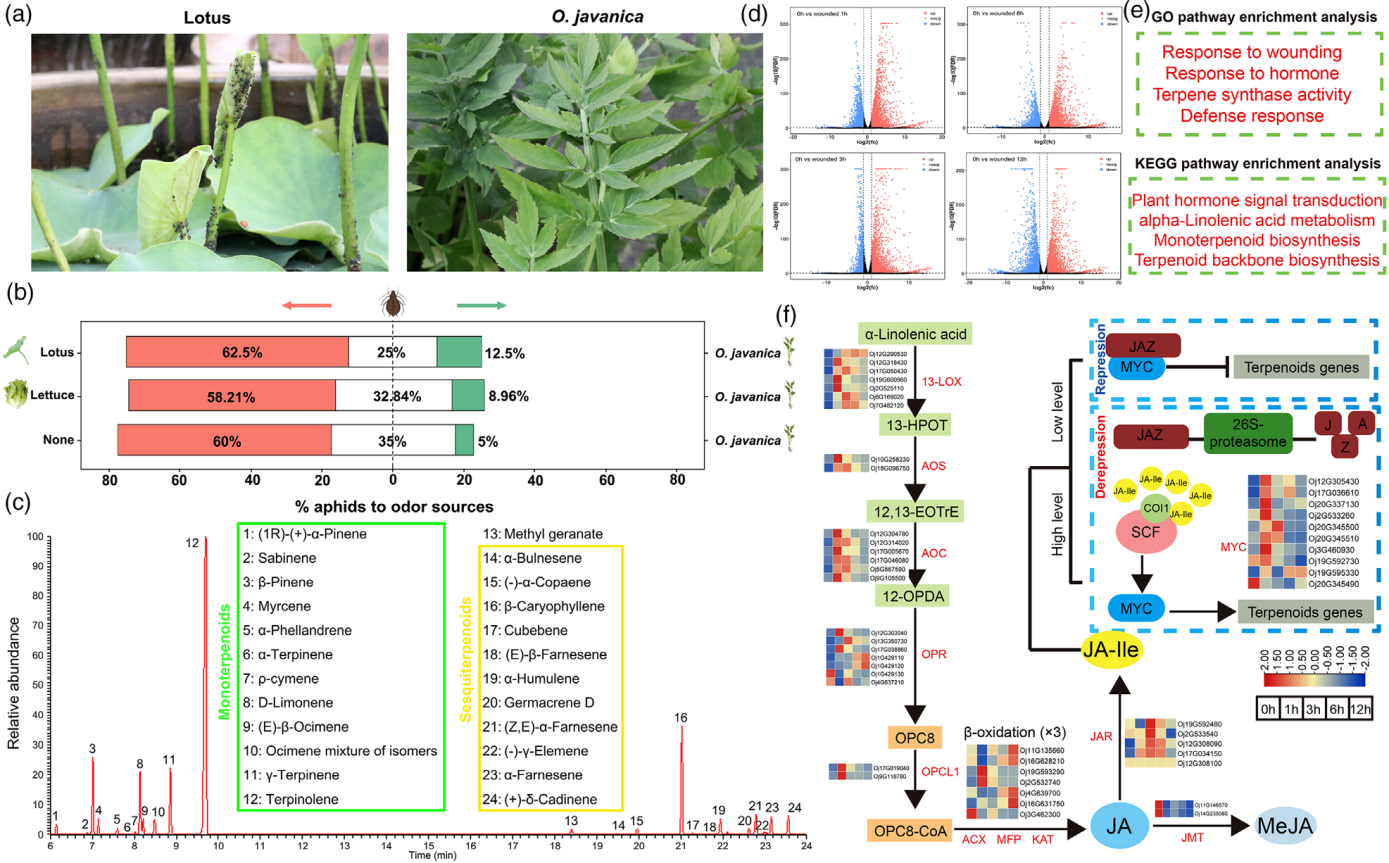
Repellent effects of *Oenanthe javanica* volatile substances on herbivorous insects. (a) Infestation of *Rhopalosiphum nymphaeae* (Linnaeus) in aquatic plants. (b) Choice of *R. nymphaeae* when offered the odour from *O. javanica* and other plants. (c) Identification of volatile substances in *O. javanica* based on GC–MS. (d) DEG analysis under wounding. (e) GO and KEGG enrichment analysis of DEGs. (f) JA biosynthesis and signal transduction pathways under wounding treatment.

Comparative genomic and evolutionary analysis showed that the WGD event resulted in numerous expansions of terpenoid‐related gene families. At the aquatic plant experimental base at Yangzhou University, the tender petioles of *N. nucifera* were infested with aphid *Rhopalosiphum nymphaeae*, but the petioles of *O. javanica* were not (Figure 3a). The responses of *R*. *nymphaeae* to different plant materials were determined to further investigate the repellent effects of *O*. *javanica* volatiles. *Rhopalosiphum nymphaeae* showed preferences for the plants of lotus and lettuce rather than for those of *O. javanica*. When placing *R*. *nymphaeae* in a tube containing *O. javanica* on one end and nothing on the other end, *O. javanica* had a repellent effect on *R. nymphaeae* (Figure 3b). To further investigate the volatile substance composition in *O. javanica*, gas chromatography–mass spectrometry (GC–MS) analysis was conducted. A total of 24 volatile substances were detected, and the main components were monoterpenoids and sesquiterpenoids (Figure 3c).

### Wounding signals mediate JA to activate volatile biosynthesis

Volatile biosynthesis was influenced by many factors, and the physical wounding was caused when herbivores invaded *O. javanica*. Perforation using dressmaker pins was conducted to simulate herbivore infestation. GC–MS analysis of wounded leaves showed that *O. javanica* rapidly releases large quantities of volatile terpenes in response to environmental stimuli (Figure S10). The time course (0, 1, 3, 6 and 12 h) transcriptome showed that numerous DEGs were detected under the wounding treatment (Figure 3d). GO and KEGG pathway analyses showed that the DEGs were significantly enriched in the response to wounding and hormone, terpene synthase activity and defense response pathways (Figure 3e). Jasmonic acid (JA) is an immunity phytohormone involved in plant defense against herbivorous insects (Hu *et al*., 2022). Time course transcriptomics indicated that the α‐linolenic acid metabolism and JA signal transduction pathways were activated after the wounding treatment. The core regulator involved in JA signal transduction, MYC TF, was significantly upregulated under mechanical damage, indicating that *O. javanica* exhibits an adaptive response to wounding signals by regulating the JA signal pathway to activate volatile terpenoid biosynthesis.

### Identification of structural genes in the terpenoid biosynthesis pathway

**Figure 4:**
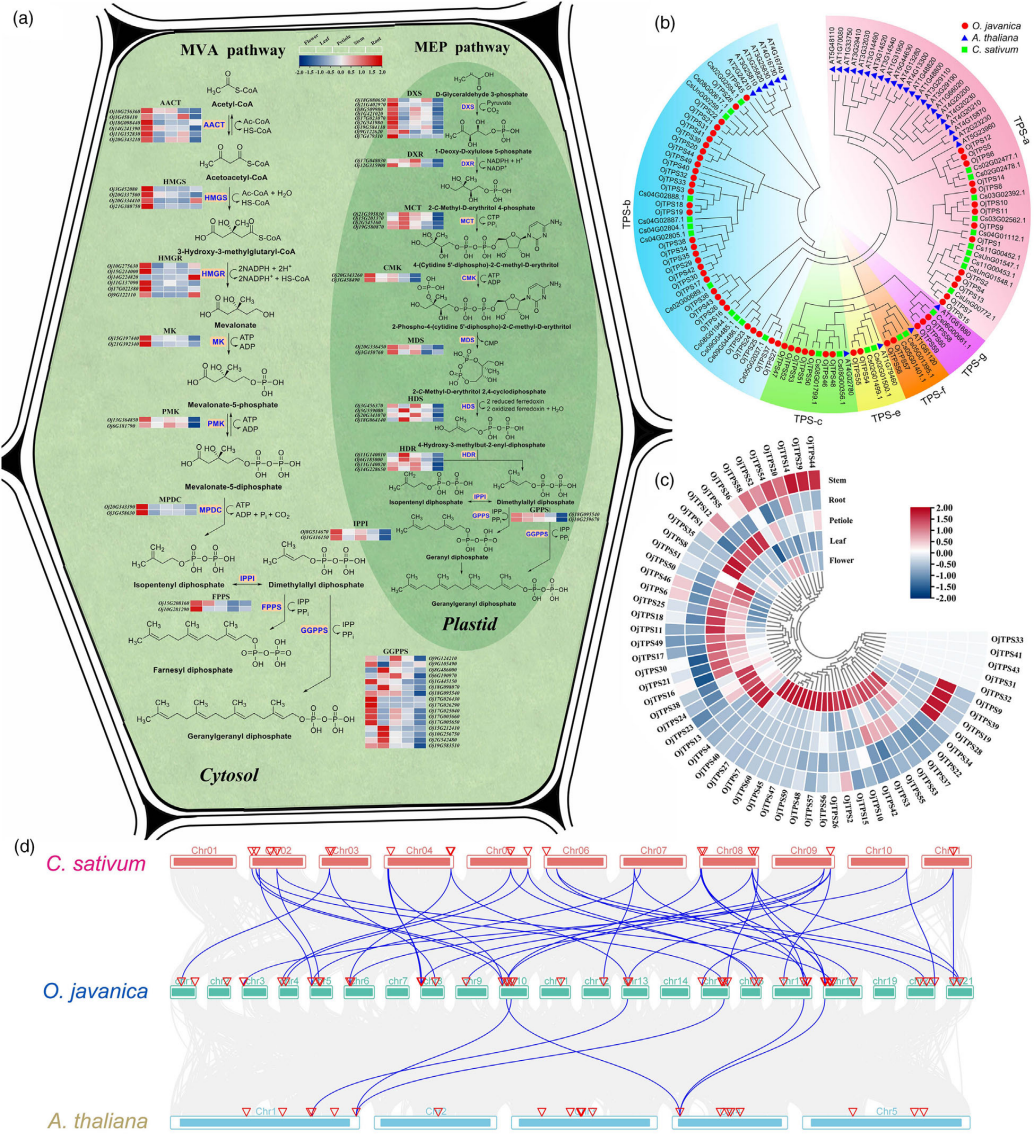
Identification of terpenoid biosynthesis genes and TPS members in *Oenanthe javanica*. (a) Identification of structural genes in terpenoid biosynthesis pathways in *O. javanica*. The five boxes with different colours next to the gene's name indicate the gene expression levels in *O. javanica* tissues. The gene expression values were calculated based on the log_2_(FPKM+1). Red and blue indicated the high and low gene expression, respectively. (b) The phylogenetic tree of TPS family members in *O. javanica*, Arabidopsis and *C. sativum*. (c) Expression heatmap of the TPS gene family in *O. javanica* tissues. (d) Collinearity analyses of TPS family members in *O. javanica*, Arabidopsis and *C. sativum*.

Considering that monoterpenoids and sesquiterpenoids are the main volatiles in *O. javanica*, the structural genes involved in terpenoid biosynthesis were identified. The terpenoid precursors isopentenyl diphosphate (IPP) and dimethylallyl diphosphate (DMAPP) were generated by the mevalonate (MVA) and methylerythritol 4‐phosphate (MEP) pathways (Vranova *et al*., 2013). Most terpenoid biosynthesis genes underwent gene expansion in *O. javanica*. Six *AACT* genes (encoding acetyl‐CoA C‐acetyltransferase), six *HMGR* genes (3‐hydroxy‐3‐methylglutary‐CoA reductase) and nine *DXS* genes (encoding 1‐deoxy‐D‐xylulose 5‐phosphate synthase) were observed in *O. javanica*, which were significantly higher than the gene copies in Arabidopsis, *D. carota* and *C. sativum* (Table S23). The expression of terpenoid biosynthesis genes in different *O. javanica* tissues was determined based on transcriptomics (Figure 4a). Most structural genes in the cytosolic MVA pathway showed high transcript abundance in flowers and low transcript abundance in roots. Notably, one of the *HMGR* genes, *Oj14G224820*, had the highest expression level in roots and may play a vital role in terpenoid biosynthesis in the roots. Expression differences among tissues will serve as an essential foundation for the subsequent study of tissue‐specific terpenoid accumulation in *O. javanica*.

### Identification of terpene synthase (TPS) gene family members

Terpene synthase (TPS) catalyses FPP, GPP and GGPP to different terpenoids in the last step of the biosynthesis process. In this study, 60 TPS members were systematically identified in the *O. javanica* T2T genome, and they were divided into TPS‐a, TPS‐b, TPS‐c, TPS‐e, TPS‐f and TPS‐g subfamilies (Figure 4b). A total of 32 TPS‐b members and 14 TPS‐a members were detected in *O. javanica*, accounting for the majority of TPS family members, and these genes are mainly responsible for monoterpenoid and sesquiterpenoid biosynthesis (Table S24 and Figure S11). The expression heatmap showed that all TPS members except *OjTPS31‐33*, *OjTPS41* and *OjTPS43* were expressed in at least one *O. javanica* tissue (Figure 4c). The number of TPS family members was significantly higher in *O. javanica* than in Arabidopsis and *C. sativum*. Collinearity analysis of TPS family members was performed in *O. javanica*, Arabidopsis and *C. sativum*. One TPS‐b gene was found to retain collinear homology in these species, with *Oj13G360250.1* in *O. javanica*, *AT1G61680.1* in Arabidopsis, *Cs04G02886.1* and *Cs09G02441.1* in *C*. *sativum*. The collinear homology of the TPS family between *O. javanica* and *C. sativum* exhibited a substantially higher degree than that observed between *O. javanica* and Arabidopsis. In addition, compared to TPS members in *C. sativum*, numerous TPS members expanded in *O. javanica* (Figure 4d). The promoter sequences of TPS members were extracted based on the T2T genome, and the *cis*‐acting elements were analysed. A large number of hormonal response elements, environmental and stress response (wounding response, stress and defense response, etc.) elements were detected (Figure S12). Time course transcriptomics showed that the TPS members were activated under wounding treatment (Table S24), which indicated that the *TPS* genes might be regulated by exogenous stimuli and play important roles in the adaptation processes of *O. javanica*.

### Functional characterization of TPS members

**Figure 5:**
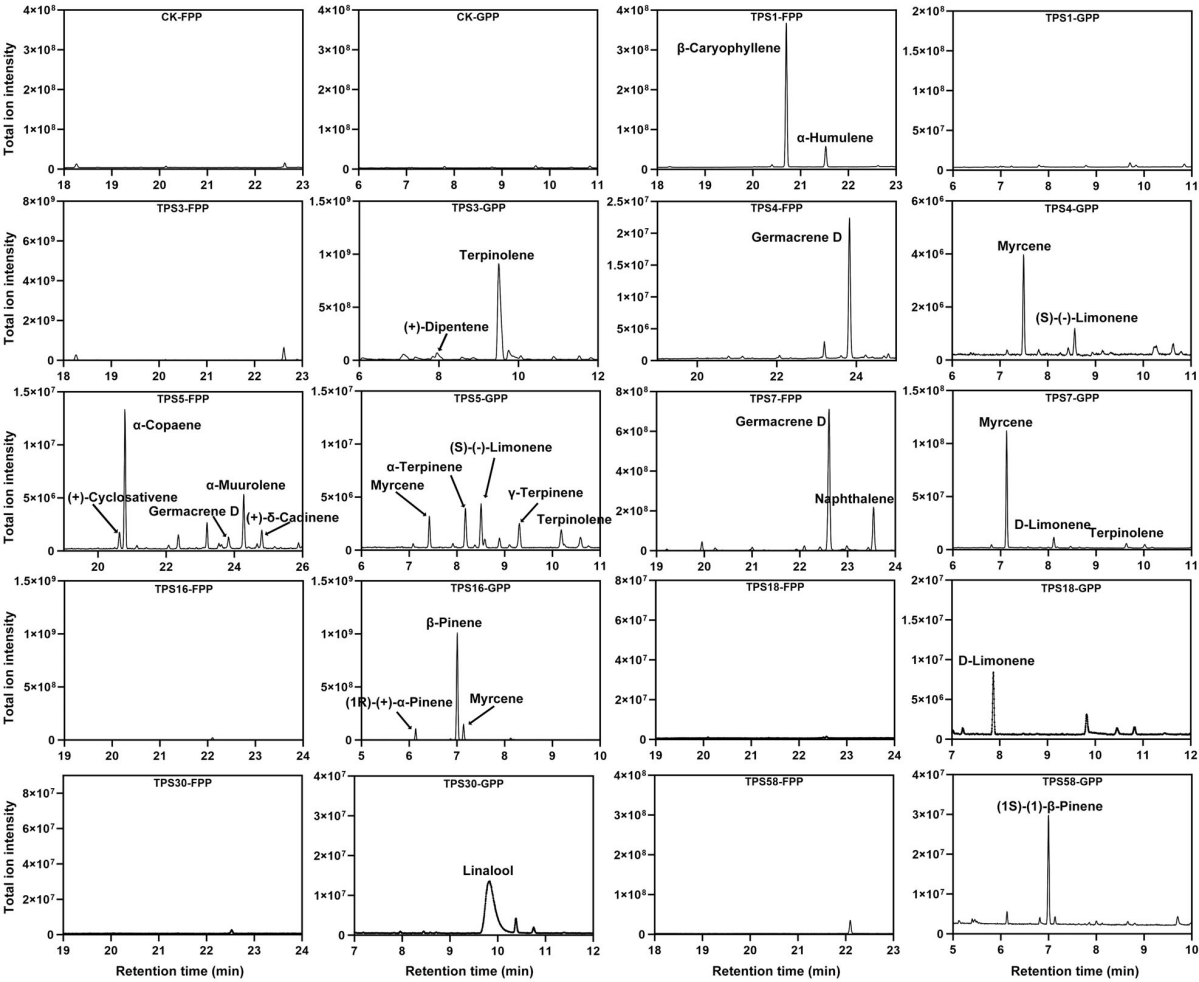
The functional verification of TPS family members. Enzymatic assay of purified empty pCold‐TF and TPS proteins incubated with FPP and GPP, respectively. The products of different reactions were detected based on GC–MS.

The diversity of terpenoids in plants is usually associated with TPS gene family members. Correlation analysis was performed between the terpenoid content and TPS expressions based on transcript abundance in different *O. javanica* tissues (Figure S13). To further characterize the TPS members responsible for terpenoid biosynthesis in *O. javanica*, candidate *OjTPS* genes with relatively high expression levels from different subfamilies (TPS‐a, TPS‐b and TPS‐g) were selected for functional identification and activity analysis. OjTPS1 and OjTPS3 were reported to be involved in the biosynthesis of β‐caryophyllene and terpinene, respectively. In this study, no products were detected from the reactions of OjTPS1 incubated with GPP or OjTPS3 incubated with FPP, indicating that these two TPS proteins only recognize specific substrates for terpenoid biosynthesis. OjTPS4, OjTPS5 and OjTPS7 in the TPS‐a subfamily were determined to be sesqui‐/mono‐TPS proteins with multiple functions. Both OjTPS4 and OjTPS7 converted FPP and GPP into germacrene D (sesquiterpenoid) and myrcene (monoterpenoid). The products of OjTPS5, including α‐copaene and other monoterpenes and sesquiterpenes, were complicated when incubated with FPP and GPP. OjTPS16, a TPS‐b subfamily member, was found to be involved in β‐pinene, (1R)‐(+)‐α‐pinene and myrcene biosynthesis. TPS‐b subfamily members OjTPS18 and OjTPS30 were identified as single‐function TPS members that catalyse GPP to D‐limonene and linalool, respectively. *In vitro* enzyme activity assays of the TPS‐g subfamily member OjTPS58 led to the conversion of GPP into (1S)‐1‐β‐pinene (Figure 5).

## Discussion

*Oenanthe javanica* is an aquatic plant belonging to the Apiaceae family, which is a medicinal food and homologous plant with multiple medical functions. It is also used as a spice due to its unique fragrance (Lu and Li, 2019). In 2021, the draft genome of *O. javanica* was published using HiSeq 2000 sequencing technology (Liu *et al*., 2021). However, the karyotype of *O. javanica* remained elusive, and the previous draft genome was not anchored to the chromosomes. With the development of long‐read sequencing technologies, the emergence of the T2T genome has facilitated genetics and molecular biology research on horticultural crops (Li *et al*., 2024). In this study, the first *O. javanica* T2T genome with the highest quality and integrity was constructed using multiple sequencing strategies.

Genome duplication events usually occur in the adaptive evolution process, resulting in novel species (Van De Peer *et al*., 2017; Wu *et al*., 2022). Comparative genomic analysis indicated that the homologous genes and expanded gene families in *O. javanica* were the most abundant among the detected species. The shared α and ω WGD events enabled Apiaceae plants to evolve into aromatic herbs with a variety of characteristics, including abundant secondary metabolite production, pharmaceutical activities and defense against pathogens and insects (Huang *et al*., 2024; Song *et al*., 2021). Notably, a recent species‐specific WGD (3.6–6.5 Mya) event was detected in *O. javanica*, which resulted in a complex karyotype, massive gene family expansion, and water adaptations not possessed by other Apioideae plants. The 2:1 ratio of the collinear regions generated by synteny analysis between *O. javanica* and other Apioideae plants further verified the species‐specific WGD in *O. javanica*.

The expansion and retention of gene families were necessary to acquire evolutionary advantages in the adaptation to environmental stimuli (Qin *et al*., 2021). The emission of volatile compounds is an effective defense strategy to resist the attack of herbivores developed during plant evolution (Qiu *et al*., 2025). The expanded gene families in *O. javanica* were mainly enriched in the terpenoid biosynthesis process, plant hormone‐related responses to wounding and secondary metabolite biosynthesis pathways. Apiaceae plants produce and release chemicals to prevent the attack of pests and pathogens (Baananou *et al*., 2013; Sahaf *et al*., 2007). In the current study, the volatile substances of *O. javanica* showed a significant repellent effect on herbivores, and the composition of volatiles was determined as mainly monoterpenoids and sesquiterpenoids. Volatile terpenoids have been proven to act as plant–insect signalling molecules to deter herbivores and attract predators during biological attacks (Xiao *et al*., 2012). The MVA and MEP pathways involved in terpenoid biosynthesis were determined based on the *O. javanica* T2T genome. The copy number of structural genes related to terpenoid biosynthesis, especially the *AACT*, *HMGS*, *HMGR* and *DXS* genes, was significantly higher in *O*. *javanica* than in Arabidopsis (Vranova *et al*., 2013). The expansion of terpenoid biosynthesis gene families greatly contributed to the volatile substance–mediated repellent effects in plant–herbivore interactions of *O*. *javanica*.

**Figure 6:**
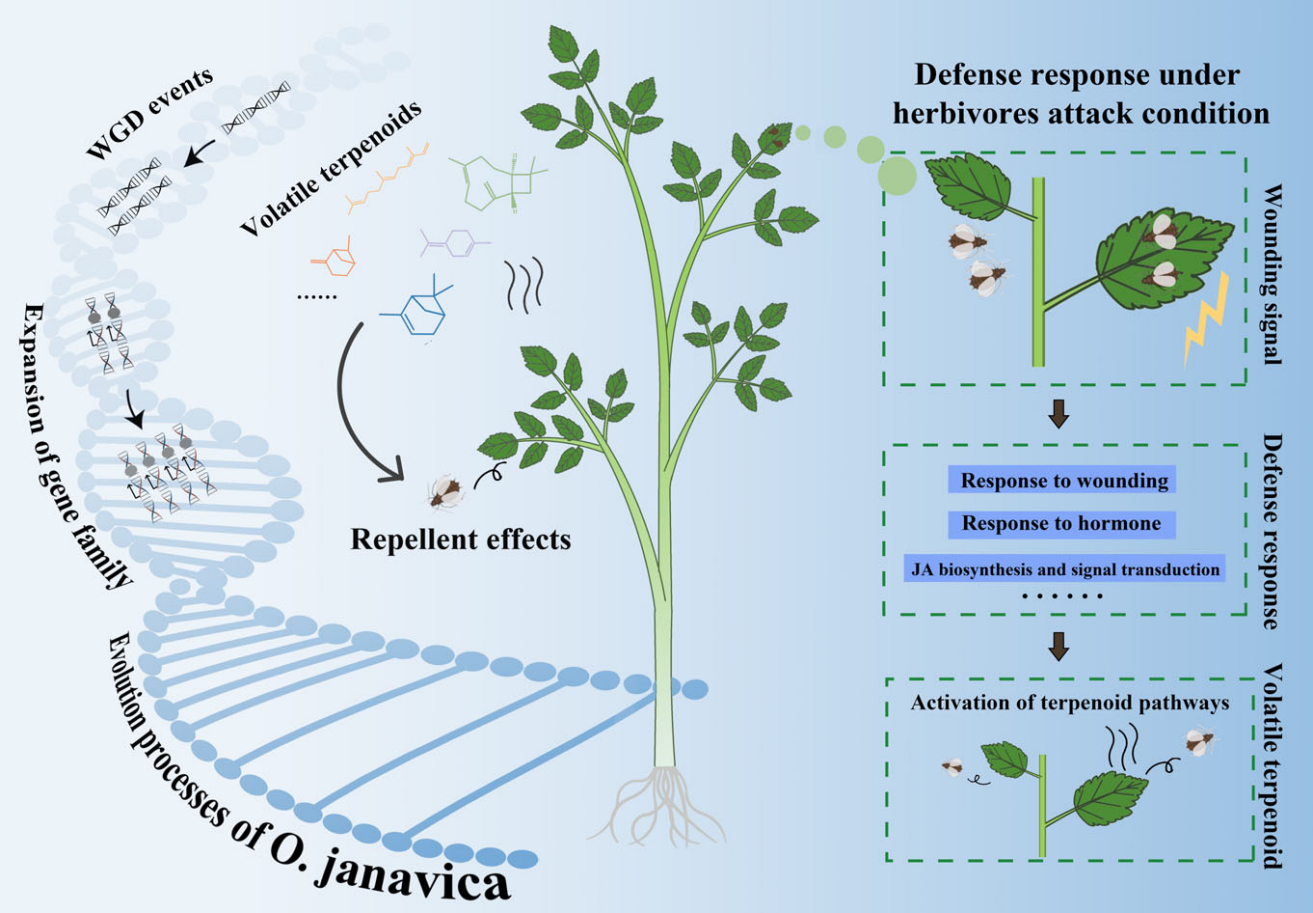
Schematic model of adaptive evolution of terpenoid domestication in *Oenanthe javanica*.

The TPS gene family plays an important role in the final stage of terpenoid biosynthesis, and the diversity of its members is an important factor affecting terpenoid abundance and diversity among species (Bao *et al*., 2020; Zheng *et al*., 2024). The TPS family members significantly expanded in *O. javanica*, but not in Arabidopsis and *C*. *sativum*. In this study, the previously reported β‐caryophyllene synthase OjTPS1 (Feng *et al*., 2023) and terpinene synthase OjTPS3 (Feng *et al*., 2024) were characterized to specifically recognize FPP or GPP for terpenoid biosynthesis. β‐pinene was a major monoterpenoid component in *O. javanica*, and its biosynthesis was shown to be related to OjTPS16 based on the enzymatic assay. In addition, the TPS‐a subfamily members OjTPS4, OjTPS5 and OjTPS7 were determined to be multifunctional enzymes serving both FPP and GPP as substrates to generate different terpenoids. Plants undergo multiple physiological responses under the attack of herbivorous insects (Steinbrenner *et al*., 2022). The expression of TPS members and other defense‐response pathways was activated under wounding treatment. Volatile substance biosynthesis and release is an important defense strategy for *O. javanica* to cope with herbivorous insects (Figure 6).

Taken together, the first high‐quality T2T genome for *O. javanica* was assembled in this study. The accumulation of terpenoids confers the repellent effects of *O. javanica* volatiles on herbivores. The biosynthesis pathway and TPS family members contributing to volatile terpenoid production were identified based on the T2T genome. In adaptive evolution processes, the species‐specific WGD event and terpene‐related gene expansion caused *O. javanica* to generate abundant terpenoids to resist the invasion of some insects. These results provide genomic resources to regulate terpenoid production and to perform future evolution, genetics, and ecology research on the herbivore‐defense mechanism of plants.

## Materials and methods

### Plant material and genome sequencing

The fresh leaf of *O. javanica* variety ‘Fuqin No. 1’ was collected for genome sequencing, which was grown in the aquatic plants base of Yangzhou University (32°39′ N, 119°42′ E). The karyotype analysis and fluorescence *in situ* hybridization were performed according to the previous described method (Wu *et al*., 2022). The high‐quality genomic DNA of *O. javanica* was extracted using the CTAB method (Allen *et al*., 2006). The genome was sequenced by Benagen Co. (Wuhan, China) with multiple strategies, including ONT ultra‐long sequencing, PacBio HiFi sequencing, Hi‐C sequencing and next‐generation sequencing. The raw data obtained from different sequencing platforms were filtered by Filtlong (v0.2.4), CCS (v6.0.0), HICUP (v0.8.0) and fastp (v0.21.0) software (Chen *et al*., 2018; Wingett *et al*., 2015).

### Genome assembly and annotation

The clean data generated by ONT ultra‐long sequencing was assembled by nextDenovo (v2.5.0) software with the parameters of read cut‐off = 1 k, blocksize = 1 g, and nextgraph options = −a 1 (Hu *et al*., 2024). Then, the assemblies of Pacbio HiFi data and integration of both ONT ultra‐long and Pacbio HiFi data were conducted using hifiasm (Cheng *et al*., 2021). Purge dups (v1.2.5) and minimap2 (Li, 2018) software was used to eliminate the haplotigs and contamination of genome assembly, respectively. The assembled genome was anchored to pseudochromosomes using the clean data of Hi‐C sequencing (Zhang *et al*., 2019). HiCExplorer was used to construct the interaction heatmap (Wolff *et al*., 2020). To obtain the high‐quality *O. javanica* T2T gapless genome, the telomeres of *O. javanica* were determined using the typical repetitive sequences (CCCTAAA/TTTAGGG at 5′ end/3′ end) as references (https://telomerase.asu.edu/sequences_telomere.html). The candidate telomeric sequences were compared to the pseudochromosomes using the nucmer (v3.1) (Kurtz *et al*., 2004). Finally, the *O. javanica* T2T gapless genome was obtained after gap‐filling and error correction using Racon (v1.6.0) with Pacbio HiFi data (Vaser *et al*., 2017).

The repetitive sequences of *O. javanica* T2T genome were determined by *de novo* prediction method (Price *et al*., 2005). The identification of TE was accomplished with the RepeatModeler (v2.0.4), LTR_FINDER, LTR harvest and LTR_retriever (v2.9.0) software (Ou and Jiang, 2018). Then, the TRF (4.09) and MISA (v2.1) software were used to predict the tandem repeats (Beier *et al*., 2017; Benson, 1999). The annotation of noncoding RNA was performed according to the typical structure with different programs, tRNAscan‐SE (v2.0.12) for tRNA prediction, RNAmmer (v1.2) for rRNA prediction, INFERNAL (v1.1.4) and Rfam (v14.9) database for snRNA and miRNA prediction (Kalvari *et al*., 2021). The gene structure of *O. javanica* T2T genome was analysed by multiple methods, including *de novo* prediction, transcriptome prediction and homology (*D. carota*, *A. graveolens*, *C. sativum* and *A. thaliana*) prediction. The results predicted by the above methods were integrated by maker (v3.01.03) software to obtain the gene structure (Holt and Yandell, 2011). The gene function was annotated based on the sequence similarity with the known databases, including GO, KEGG, Nr, Pathway, Uniprot, KOG, Pfam and Interpro databases.

### Genome evolution

The *O. javanica* T2T genome and 14 other families' plant genomes (*A. sinensis*, *A. graveolens*, *A. thaliana*, *C. sativum*, *D. carota*, *H. macrophylla*, *L. sativa*, *L. japonica*, *M. truncatula*, *N. nucifera*, *O. sativa*, *S. lycopersicum*, *S. tuberosum* and *V. vinifera*) were selected to investigate the evolutionary relationship according to previous studies (Cao *et al*., 2024a,b; Sun *et al*., 2024). The orthologous gene families were identified from the 15 species by OrthoFinder software (v2.4) (Emms and Kelly, 2019). The sequence alignments of single‐copy gene families were conducted by Muscle (v3.8.31) software (Edgar, 2004), and the phylogenetic tree of 15 species was constructed by RAxML (v8.2.10) software. CAFÉ (v3.1) software was used to determine the extraction and expansion of gene families according to the gene family and phylogenetic tree. The function enrichment of extracted and expanded gene families was analysed based on the KEGG and GO databases. The WGD event was determined by *Ks* analysis of collinear gene pairs, and the density map in different species was constructed by ggplot2 (v2.2.1). Collinearity analysis of *O. javanica* with *C. sativum* and *D. carota* was performed by using last (v1170) and JCVI (0.9.13) software (Tang *et al*., 2008). The karyotype evolution was analysed based on the collinearity relationship of AEK with other Apiaceae plants (Murat *et al*., 2017).

### Olfactory response of herbivorous insects to *O. javanica* and other plants

Many aquatic plants, including *O. javanica* and *N. nucifera*, were preserved and propagated in the aquatic plants experimental base of Yangzhou University. In the spring, the young petiole of *N. nucifera* was heavily infested with *R. nymphaeae*, while the *O. javanica* in the same environment was not infected. The olfactory response of *R. nymphaeae* to *O. javanica* and other plants was performed in the round tube with a hole in the middle (diameter, 5 cm; length, 120 cm). *O. javanica* and other plants were placed at each end of the tube, and the aphids were put into the tube through the middle hole. The behavioural response of aphids to different plants was recorded after 20 min. To determine the composition of volatiles of *O. javanica*, solid phase microextraction‐gas chromatography‐mass spectrometry (SPME‐GC–MS) assay was performed as previously described (Feng *et al*., 2022).

### Wounding treatment and time‐course transcriptome analysis

The 40‐day‐old *O. javanica* without mechanical damage was used to conduct the wounding treatment, which was cultivated in the light incubator (16 h of light at 25 °C and 8 h of dark at 18 °C). The wounding treatment was performed y using dressmaker pins to puncture the leaves of *O. javanica* (Myers *et al*., 2023). The wounded and healthy leaves were collected and placed in the 20 mL vial to determine the emission of volatile terpenoids under mechanical damage treatment. In addition, the *O. javanica* leaves were collected at 0, 1, 3, 6 and 12 h after the wounding treatment for time‐course transcriptome analysis. The samples were sent to GENEDENOVO Co. (Guangzhou, China) for transcriptome sequencing.

To investigate the gene transcription level in different tissues, the flowers, petioles, leaf blades, roots and stems of *O. javanica* were sampled at 8 days after flowering. Each sample with three biological replicates was immediately frozen with liquid nitrogen and sent to Biomarker Co. (Beijing, China) for transcriptome sequencing. A total of 96.22 Gb clean data were obtained (Table S25), and these data were mapped to the current *O. javanica* T2T genome by HISAT2 tools (Kim *et al*., 2019).

### Analysis of terpenoid biosynthesis genes and terpene synthase (TPS)

The terpenoids biosynthesis genes in the MVA and MEP pathways were identified from the *O. javanica* genome based on the genome annotation and sequence similarity with Arabidopsis proteins (Vranova *et al*., 2013). The typical Pfam domains (PF01397 and PF03036) of the TPS gene family were used to search the TPS members in *O. javanica*. The phylogenetic tree of TPS members in different species was constructed by MEGA7.0 software (Kumar *et al*., 2016). The TPS members of *O. javanica* were classified based on the phylogenetic relationships with the previous classifications in Arabidopsis and *C. sativum* (Aubourg *et al*., 2002; Song *et al*., 2019). The gene expression heatmap and collinearity analyses of TPS family members in *O. javanica*, Arabidopsis and *C. sativum* were conducted by TBtools (Chen *et al*., 2020).

### Protein purification and *in vitro* enzymatic assay

Based on the classification and transcripts in different tissues of *O. javanica*, the candidate members in the TPS‐a, TPS‐b and TPS‐g subfamilies that are involved in monoterpenes and sesquiterpenes biosynthesis were selected for functional identification (Chen *et al*., 2011). The *TPS* genes were cloned from cDNA of *O. javanica* and then constructed into the expression vector pColdTF (GenBank No. AB213654) between *Bam* HI and *Sal* I sites. The recombinant pColdTF vectors were transformed into *Escherichia coli* BL21 (DE3), and the TPS proteins were induced with 1 mM of isopropyl β‐D‐thiogalactoside (IPTG). The TPS proteins were purified using the His‐Tagged Protein Purification Kit (CWBIO Co., Shanghai, China) according to the manufacturer's instructions. The purified TPS proteins were further determined by SDS‐PAGE assay. The *in vitro* enzymatic assay of purified TPS proteins was performed with terpenoid substrates, geranyl pyrophosphate (GPP) and farnesyl pyrophosphate (FPP). The terpenoid product was detected by GC–MS following our previous procedures (Feng *et al*., 2023). The primers used for gene cloning and vector construction were listed in (Table S26).

## Funding

This study was financially supported by the National Natural Science Foundation of China (32102368), China Agriculture Research System (CARS‐24) and Jiangsu seed industry revitalization project (JBGS[2021]017).

## Conflict of interest

The authors declare that they have no conflicts of interest.

## Author contributions

KF and LJL initiated and designed the research. KF, JLL, NS, ZQZ, ZYY, HL, CY, JPZ and SPZ performed the experiments. KF and JLL analysed the data. KF and LJL contributed reagents/materials/analysis tools. KF wrote the manuscript. PW and LJL revised the manuscript. All authors read and approved the final manuscript.

## Supporting information


**Figure S1** Estimation of *Oenanthe javanica* genome size by *K*‐mer analysis. X‐axis shows *K*‐mer = 19 depth. Y‐axis shows *K*‐mer frequency. The genome size was measured as 951.53 Mb.
**Figure S2** The distribution of gene length, CDS length, exon length and intron length in *Oenanthe javanica* and other plants.
**Figure S3** The gene function annotation of *Oenanthe javanica* T2T genome.
**Figure S4** Chromosomal visualization of collinear genes with *Ks* values < 0.1.
**Figure S5** GO enrichment analysis of collinear gene pairs (*Ks* <0 .1) in *Oenanthe javanica*.
**Figure S6** KEGG enrichment analysis of collinear gene pairs (*Ks* < 0.1) in *Oenanthe javanica*.
**Figure S7** Collinearity and synteny analyses of *Oenanthe javanica* with other Apiaceae plants (*C. sativum* and *D. carota*).
**Figure S8** The syntenic depth ratio analyses of *Oenanthe javanica* vs *Daucus carota* and *Coriandrum sativum*.
**Figure S9** The karyotype analysis of Apiaceae from ancestral eudicot karyotype (AEK).
**Figure S10** GC–MS analysis of *Oenanthe javanica* leaves under mechanical damage treatment.
**Figure S11** The chromosomal location of TPS family members in *Oenanthe javanica*.
**Figure S12** Cis‐acting element analysis of the promoters of TPS gene family.
**Figure S13** Correlation analysis between terpenoids from different tissues and the expressions of different TPS members.

**Table S1** Statistics data of K‐mer analysis.
**Table S2** Statistics data of ONT ultra‐long sequencing.
**Table S3** Statistics data of PacBio HiFi sequencing.
**Table S4** Statistics data of NGS sequencing.
**Table S5** Statistics data of Hi‐C sequencing.
**Table S6** Statistics of chromosome length based on Hi‐C analysis.
**Table S7** Statistics of gap number in chromosomes.
**Table S8** The consistency evalues of NGS, ONT ultra‐long data and Pacbio HiFi data.
**Table S9** Statistics of QV values in different chromosomes.
**Table S10** Statistics of busco analysis.
**Table S11** Telomere identification results.
**Table S12** Centromere identification results.
**Table S13** Statistics of TE in *Oenanthe javanica* T2T genome.
**Table S14** Statistics of tandem repeats in *Oenanthe javanica* T2T genome.
**Table S15** Annotation of ncRNA in *Oenanthe javanica* T2T genome.
**Table S16** Annotation of gene structure in *Oenanthe javanica* T2T genome.
**Table S17** Annotation of gene funtion in *Oenanthe javanica* T2T genome.
**Table S18** The gene number of each family in the *Oenanthe javanica* T2T genome and other 14 plants.
**Table S19** GO enrichment analysis of expanded gene families in *Oenanthe javanica* T2T genome.
**Table S20** KEGG enrichment analysis of expanded gene families in *Oenanthe javanica* T2T genome.
**Table S21** GO enrichment analysis of collinear gene pairs (*Ks* <0.1) in *Oenanthe javanica* T2T genome.
**Table S22** KEGG enrichment analysis of collinear gene pairs (*Ks* <0.1) in *Oenanthe javanica* T2T genome.
**Table S23** The structural genes of terpenoid biosynthesis pathways in *Oenanthe javanica*, *Daucus carota* and *Coriandrum sativum*.
**Table S24** The TPS gene identified from *Oenanthe javanica* T2T genome.
**Table S25** Statistics data of NGS sequencing in different tissues of *Oenanthe javanica*.
**Table S26** Primer sequence of gene cloning and vector construction.

## Data Availability

The genome sequences and raw RNA‐seq data described in this article were submitted to NCBI (https://www.ncbi.nlm.nih.gov/) under the BioProject accession number PRJNA1201620.
