# Supplementary material for: Telomere‐to‐telomere genome assembly reveals insights into the adaptive evolution of herbivore‐defense mediated by volatile terpenoids in Oenanthe javanica
Source: Plant Biotechnol J. 2025 Mar 20;23(6):2346–57. doi: 10.1111/pbi.70062 (PMC12120883; doi:10.1111/pbi.70062)
Supplement: Supplementary file 1 — Figure S1 Estimation of Oenanthe javanica genome size by K‐mer analysis. X‐axis shows K‐mer = 19 depth. Y‐axis shows K‐mer frequency. The genome size was measured as 951.53 Mb. Figure S2 The distribution of gene length, CDS length, exon length and intron length in Oenanthe javanica and other plants. Figure S3 The gene function annotation of Oenanthe javanica T2T genome. Figure S4 Chromosomal visualization of collinear genes with Ks values < 0.1. Figure S5 GO enrichment analysis of collinear gene pairs (Ks <0 .1) in Oenanthe javanica. Figure S6 KEGG enrichment analysis of collinear gene pairs (Ks < 0.1) in Oenanthe javanica. Figure S7 Collinearity and synteny analyses of Oenanthe javanica with other Apiaceae plants (C. sativum and D. carota). Figure S8 The syntenic depth ratio analyses of Oenanthe javanica vs Daucus carota and Coriandrum sativum. Figure S9 The karyotype analysis of Apiaceae from ancestral eudicot karyotype (AEK). Figure S10 GC–MS analysis of Oenanthe javanica leaves under mechanical damage treatment. Figure S11 The chromosomal location of TPS family members in Oenanthe javanica. Figure S12 Cis‐acting element analysis of the promoters of TPS gene family. Figure S13 Correlation analysis between terpenoids from different tissues and the expressions of different TPS members. [file PBI-23-2346-s002.docx]

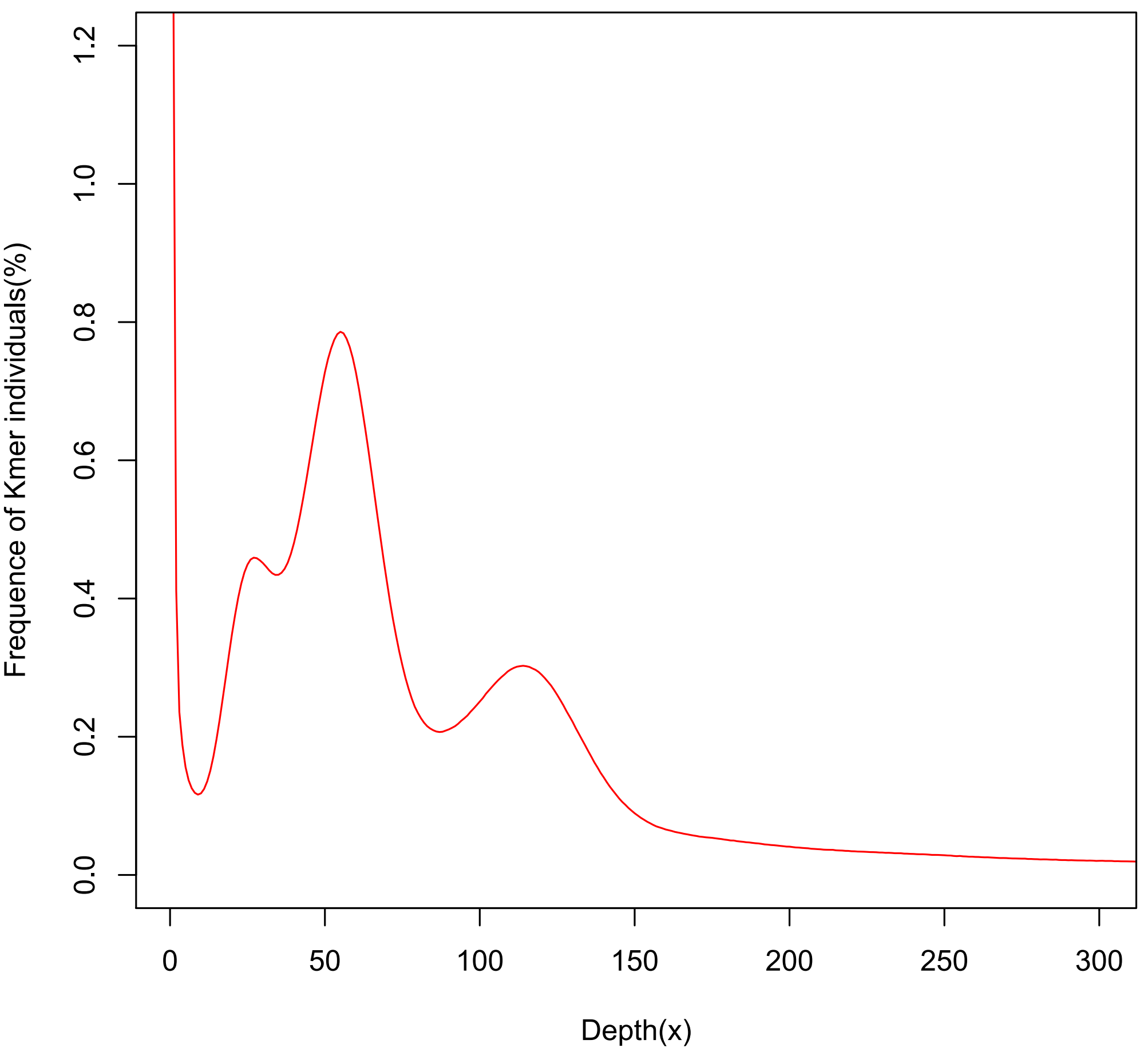


**Figure S1 Estimation of *Oenanthe javanica* genome size by *K*-mer analysis**

X axis shows *K*-mer = 19 depth. Y axis shows *K*-mer frequency. The genome size was measured as 951.53 Mb.


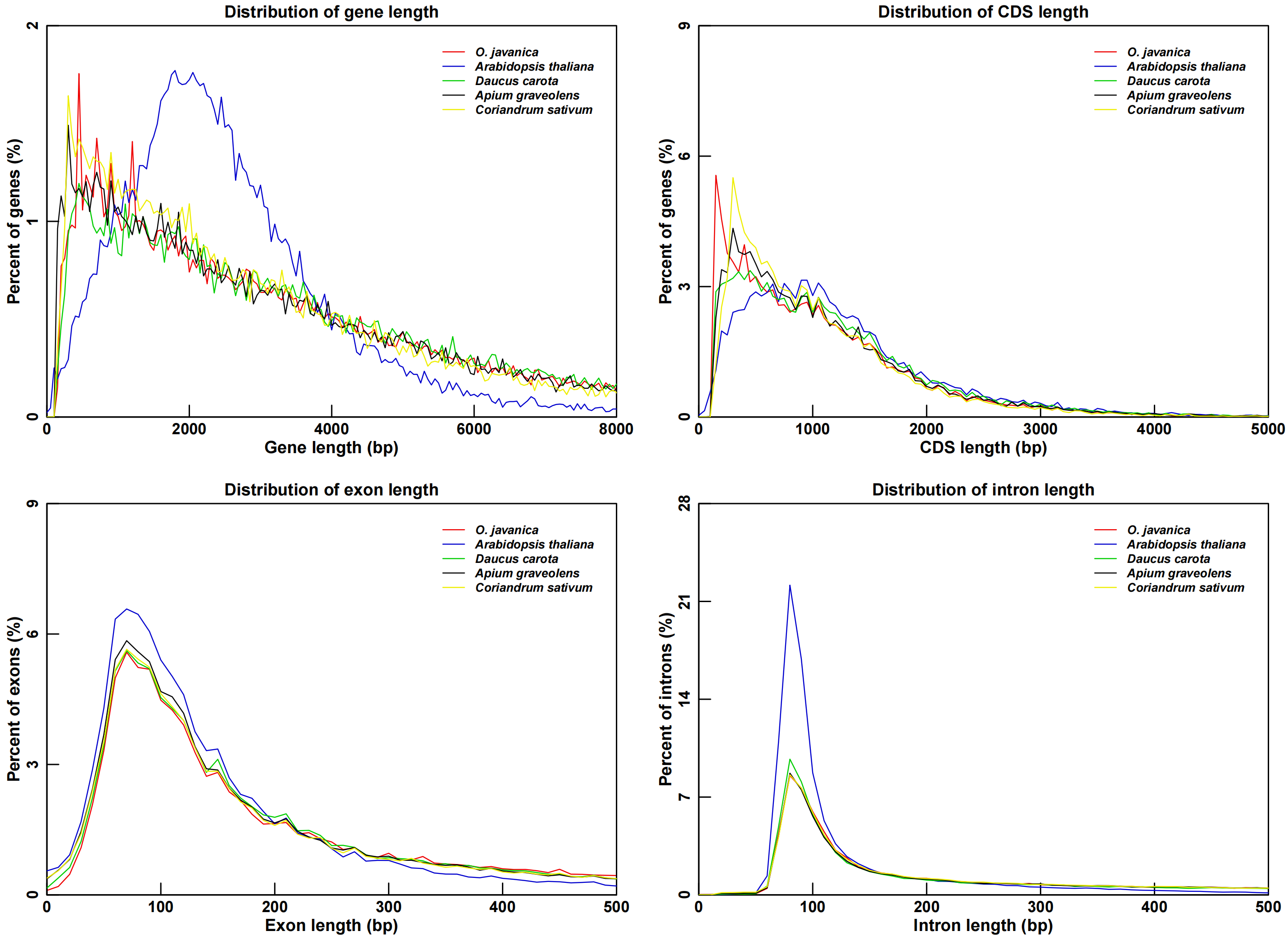


**Figure S2 The distribution of gene length, CDS length, exon length, and intron length in** ***Oenanthe javanica* and other plants**


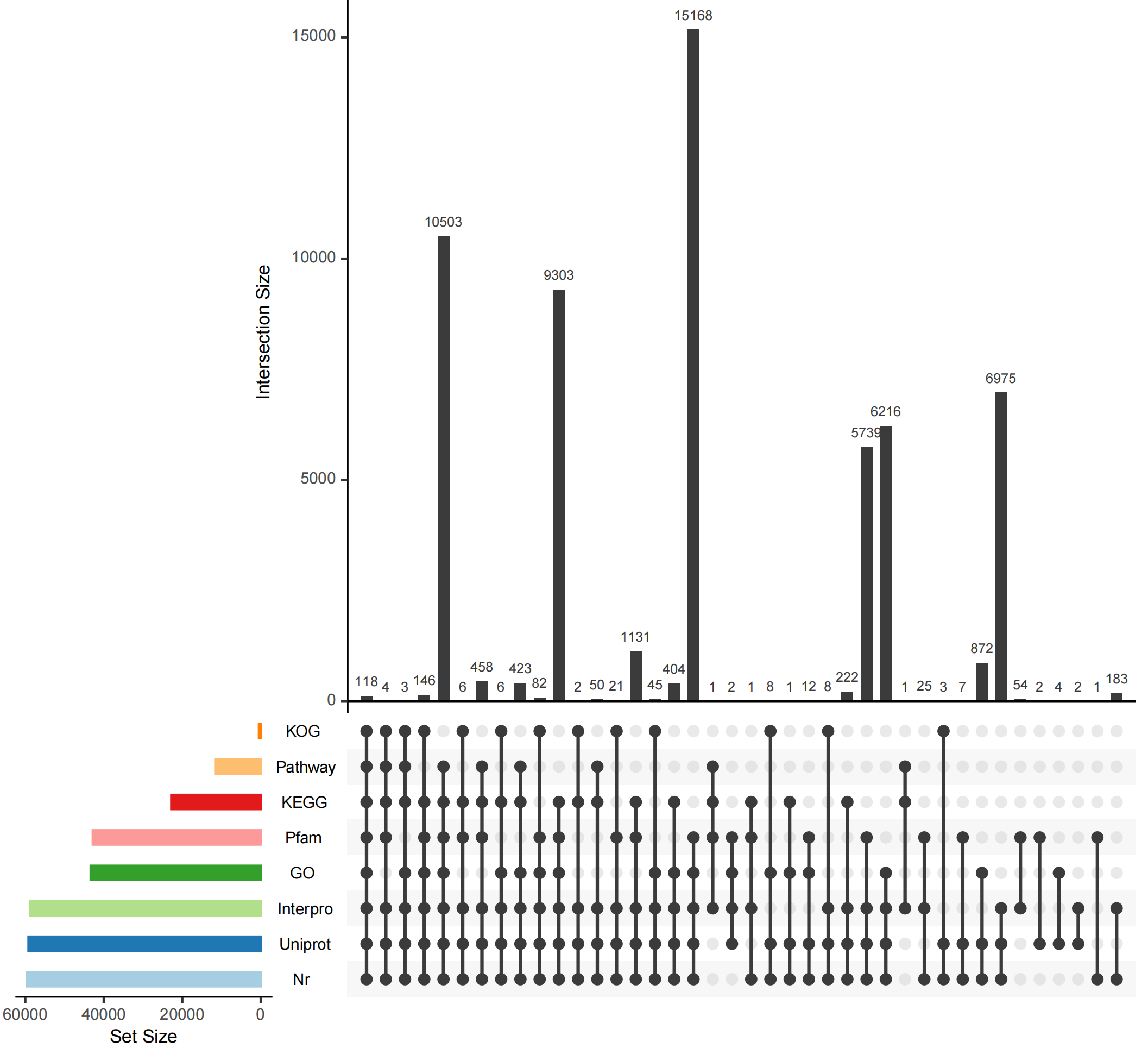


**Figure S3 The gene function annotation of *O. javanica* T2T genome**


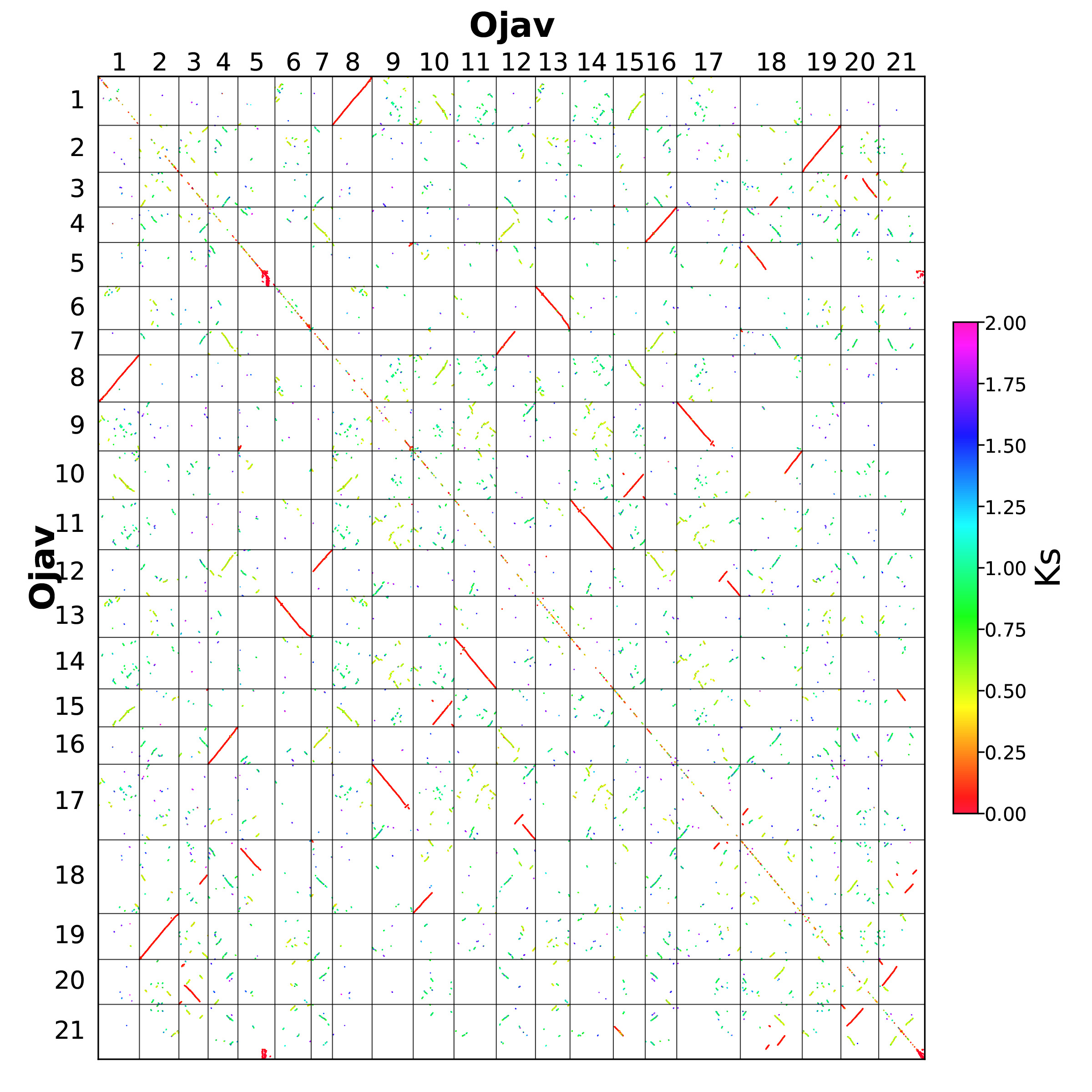


**Figure S4 Chromosomal visualization of collinear genes with *Ks* values less than 0.1.**


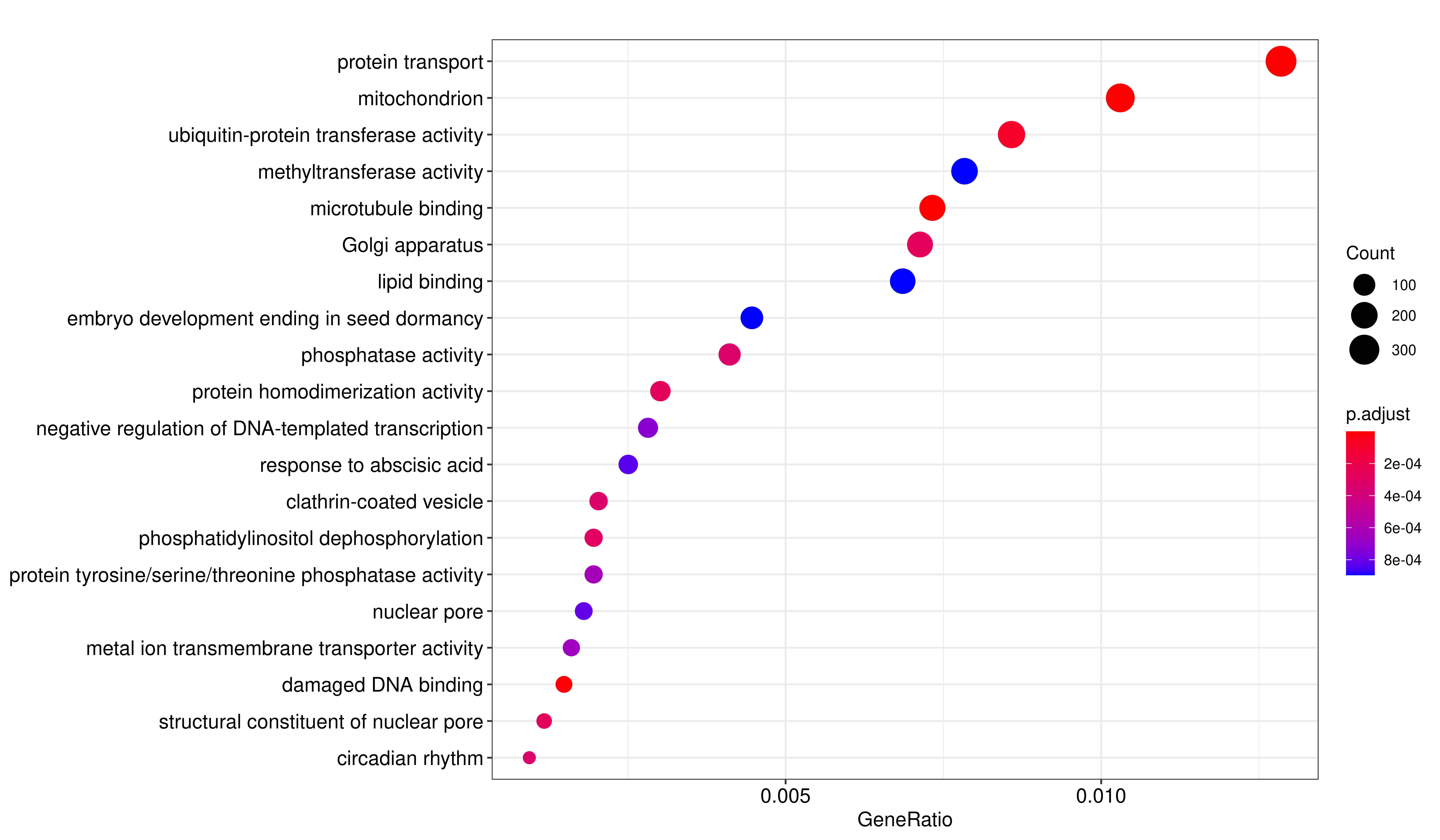


**Figure S5 GO enrichment analysis of collinear gene pairs (*Ks* < 0.1) in *O. javanica*.**


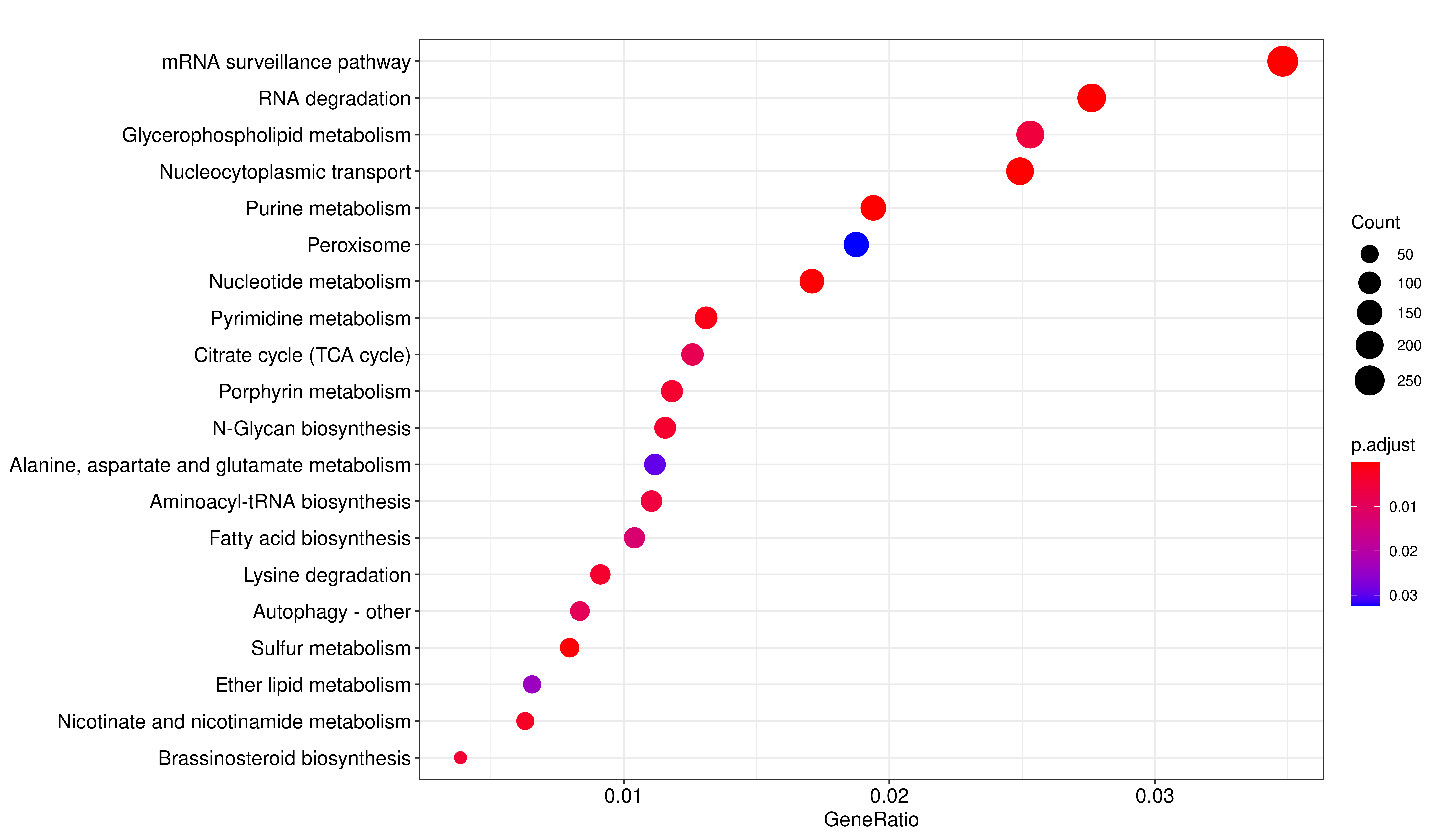


**Figure S6 KEGG enrichment analysis of collinear gene pairs (*Ks* < 0.1) in *O. javanica*.**


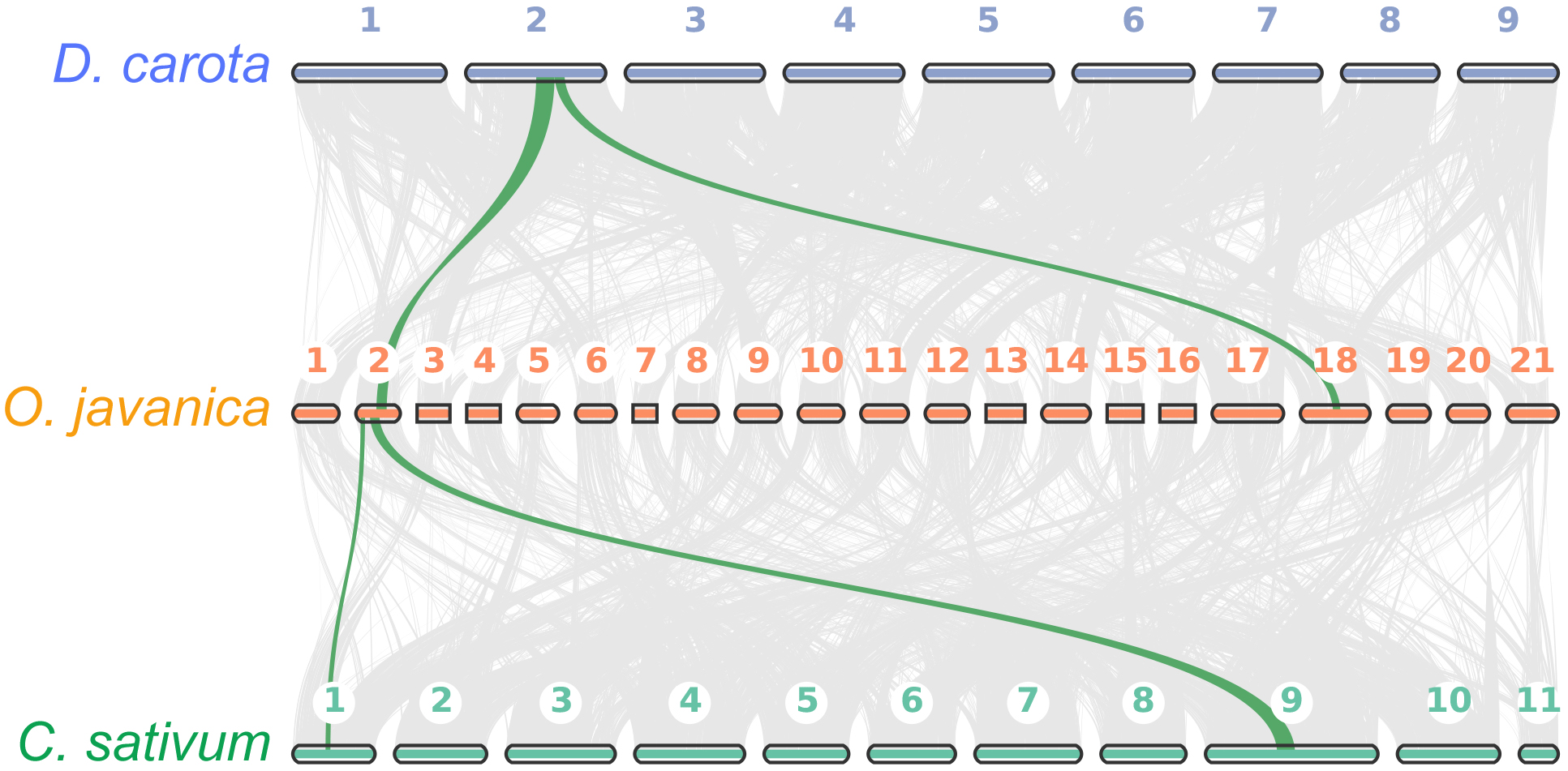


**Figure S7 Collinearity and synteny analyses of *O. javanica* with other Apiaceae plants (*C. sativum* and *D. carota*).**


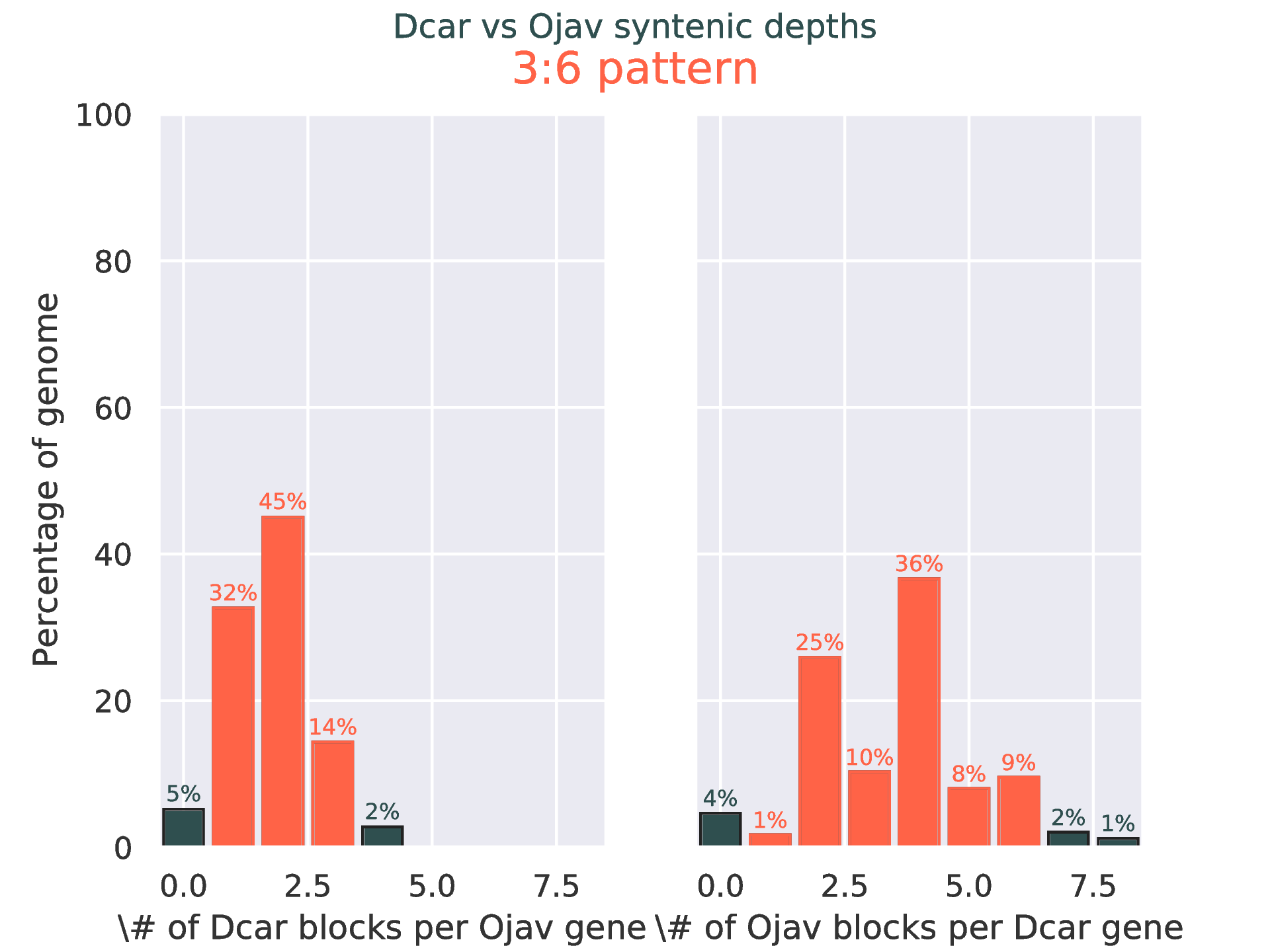

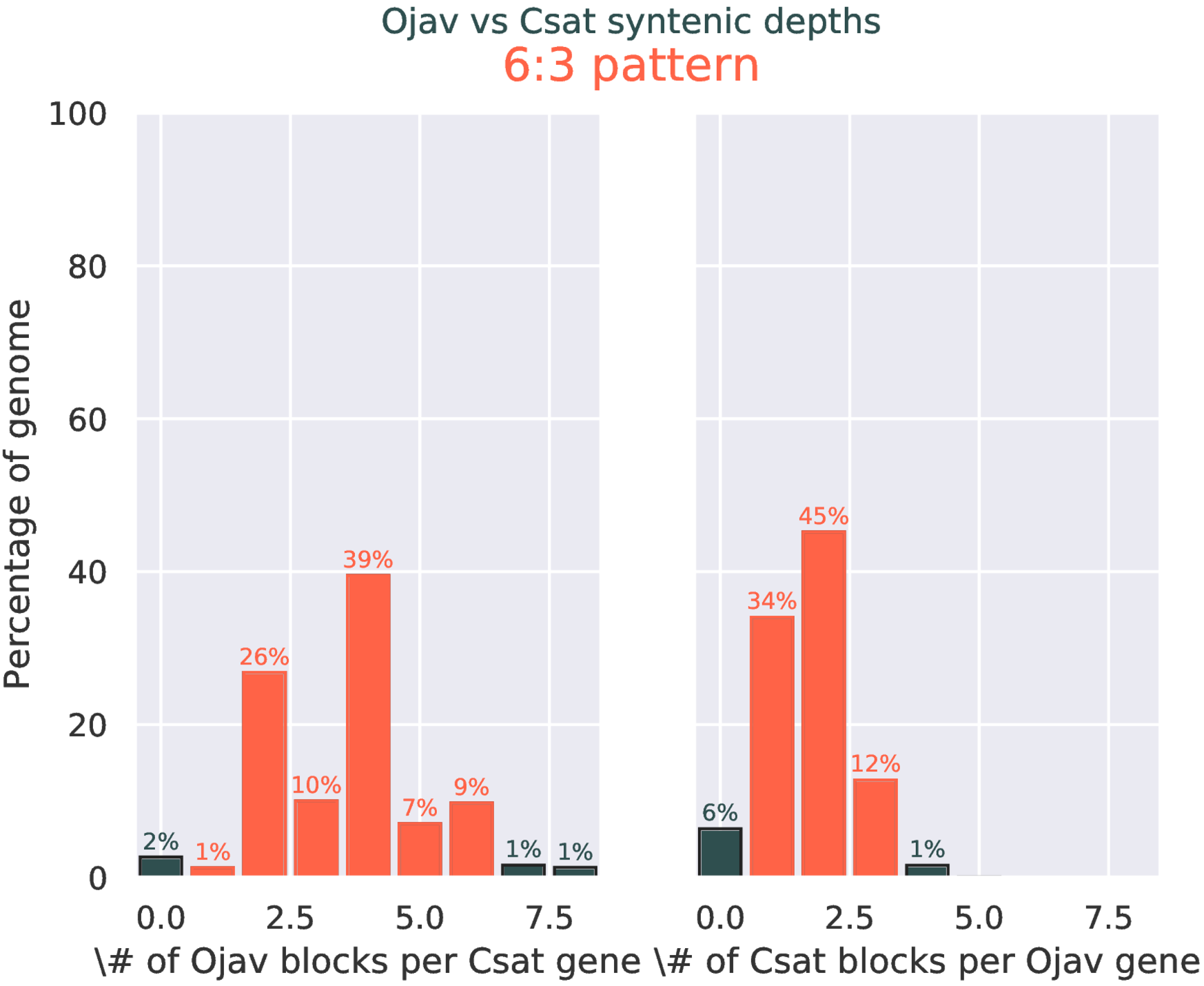


**Figure S8 The syntenic depth ratio analyses of *Oenanthe javanica* vs *Daucus carota* and *Coriandrum sativum.***


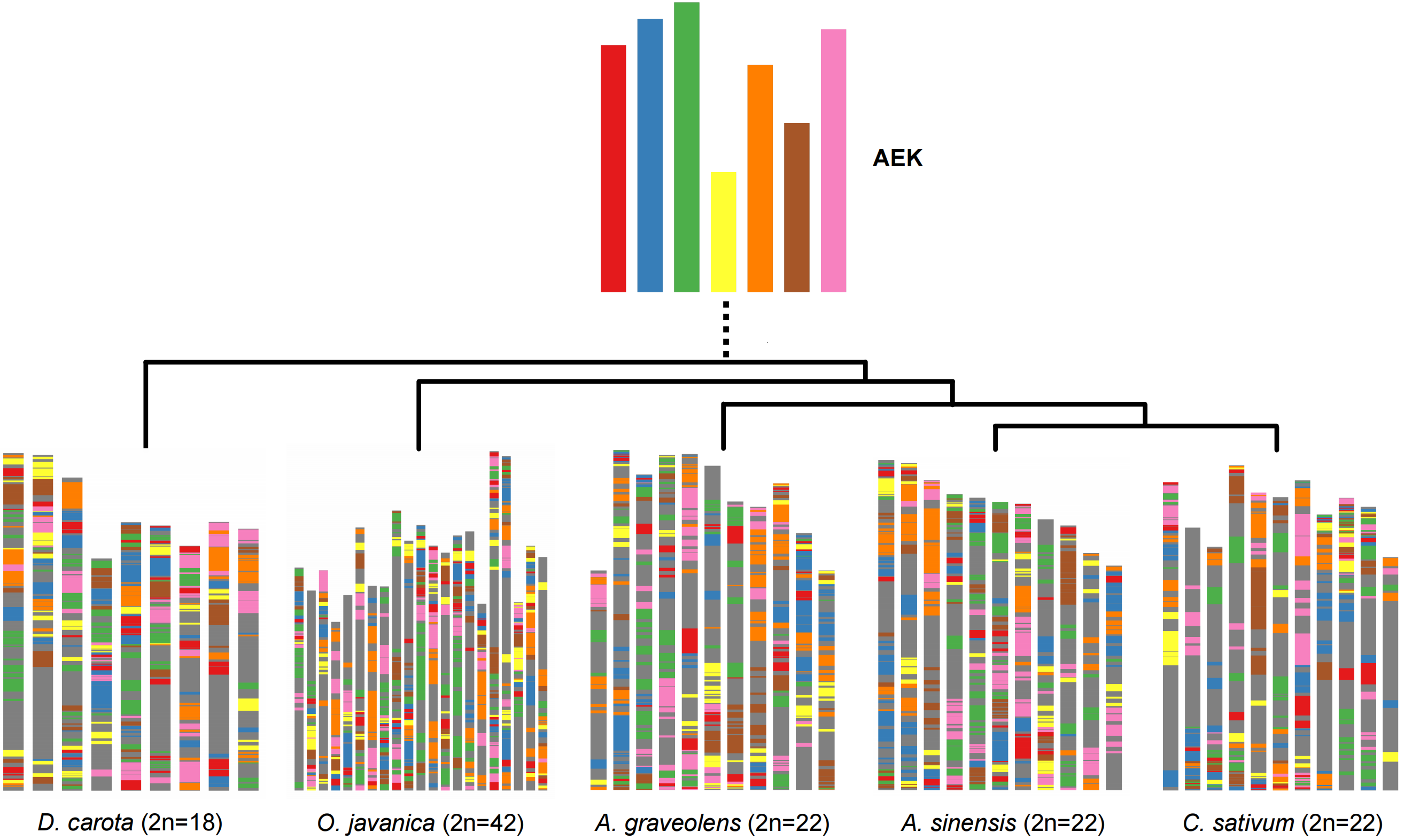


**Figure S9 The karyotype analysis of Apiaceae from ancestral eudicot karyotype (AEK).**


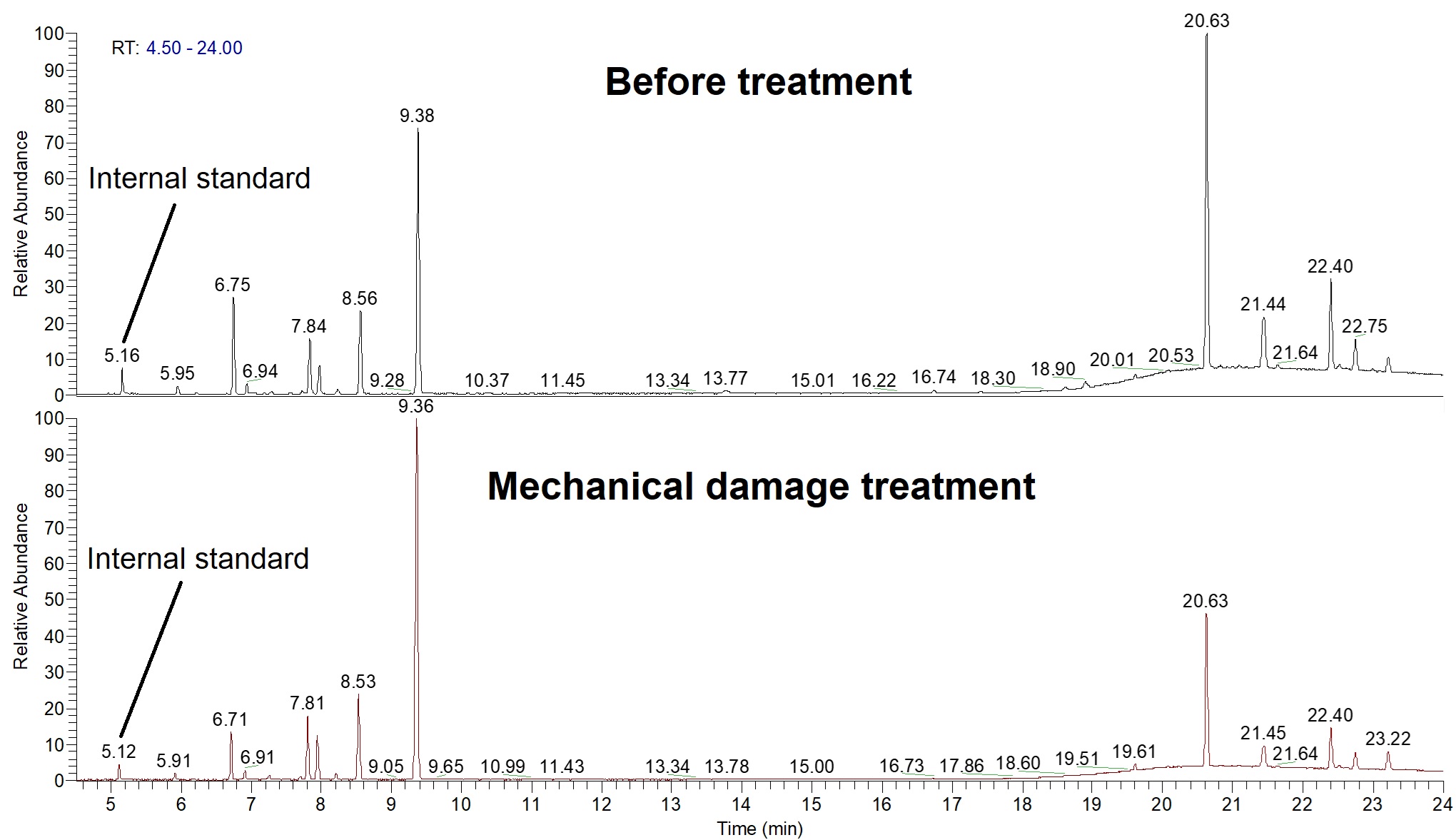


**Figure S10** **GC-MS analysis of *Oenanthe javanica* leaves under mechanical damage treatment.**





**Figure S11 The chromosomal location of TPS family members in *Oenanthe javanica.***

**Figure S12 Cis-acting element analysis of the promoters of TPS gene family.**


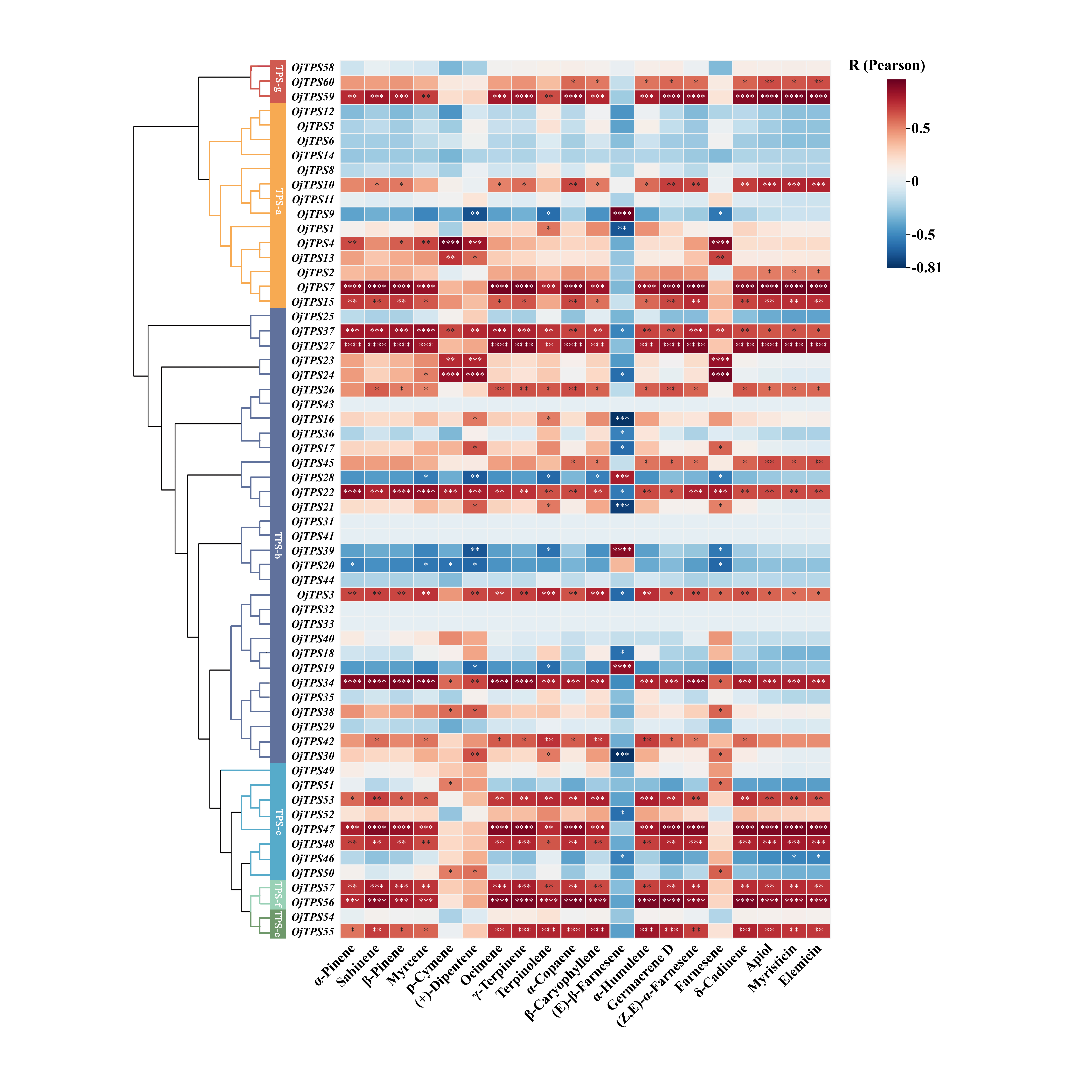


**Figure S13** **Correlation analysis between** **terpenoids from different tissues and the expressions of different TPS members.**
